# Supplementary material for: Collagen depletion by pirfenidone enhances antitumor effect of oncolytic adenovirus against peritoneal metastases of gastric cancer
Source: Mol Ther Oncol. 2025 Sep 2;33(4):201045. doi: 10.1016/j.omton.2025.201045 (PMC12859411; doi:10.1016/j.omton.2025.201045)
Supplement: Document S1. Figures S1–S7 and Table S1 and S2 [file mmc1.pdf]

OMTON, Volume *33*

Supplemental information

Collagen depletion by pirfenidone enhances antitumor effect of oncolytic adenovirus against peritoneal metastases of gastric cancer

Tomohiro Okura, Satoru Kikuchi, Hiroshi Tazawa, Yu Mikane, Nobuhiko Kanaya, Ema Mitsui, Yuta Une, Kunitoshi Shigeyasu, Toshiaki Ohara, Shinji Kuroda, Kazuhiro Noma, Junko Ohtsuka, Rieko Ohki, Shunsuke Kagawa, Yasuo Urata, and Toshiyoshi Fujiwara

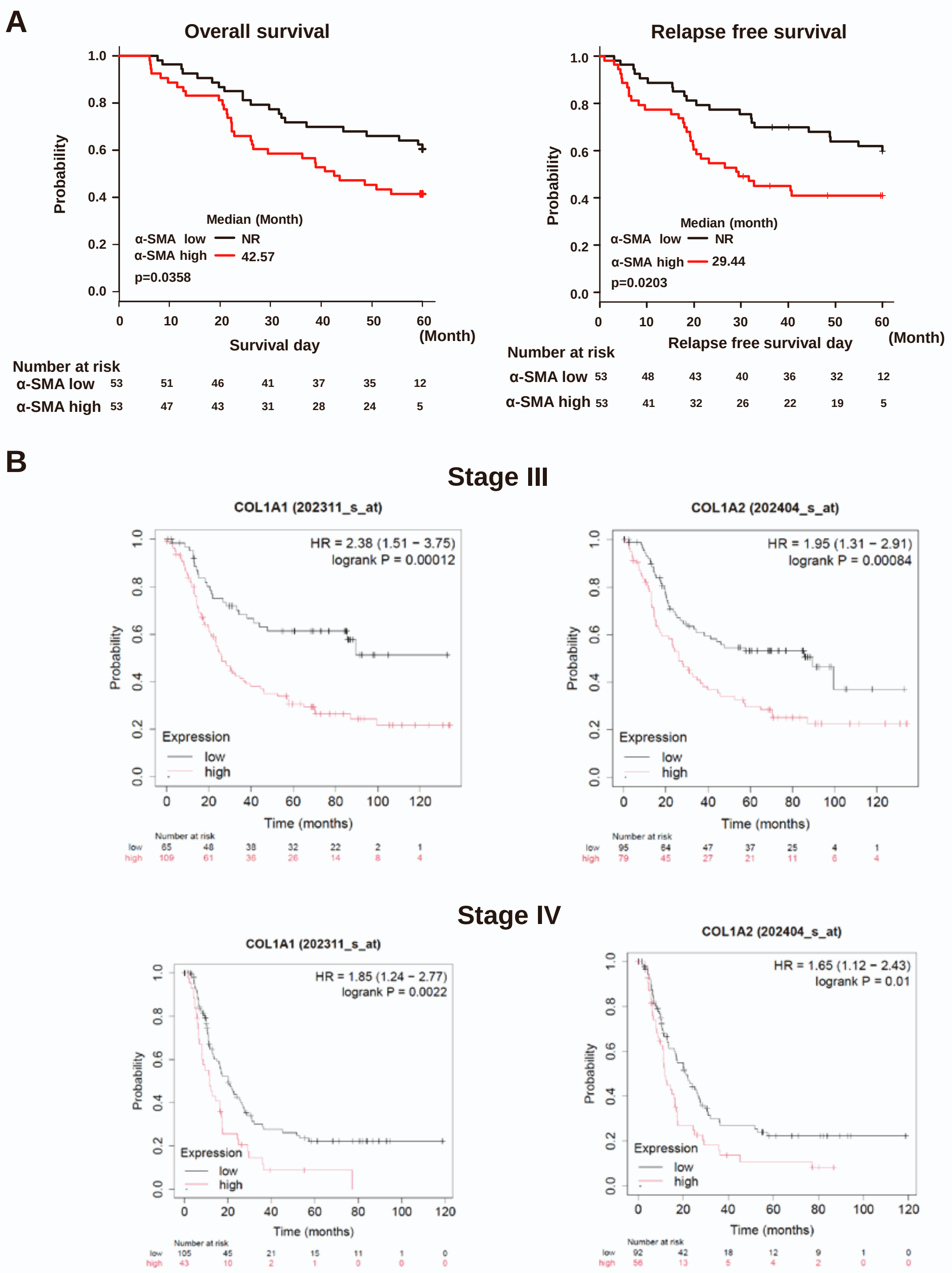

Fig. S1

A) Overall survival and relapse free survival curve according to α-SMA expression (high or low) in the primary gastric cancer. The high α-SMA expression group showed significantly worse prognosis (log rank test).

B) The relationship between the RNA expression levels of Col1A1 or Col1A2 and overall survival estimates in patients for Stage III and Stage IV gastric cancer patients was evaluated using Kaplan–Meier plotter datasets.

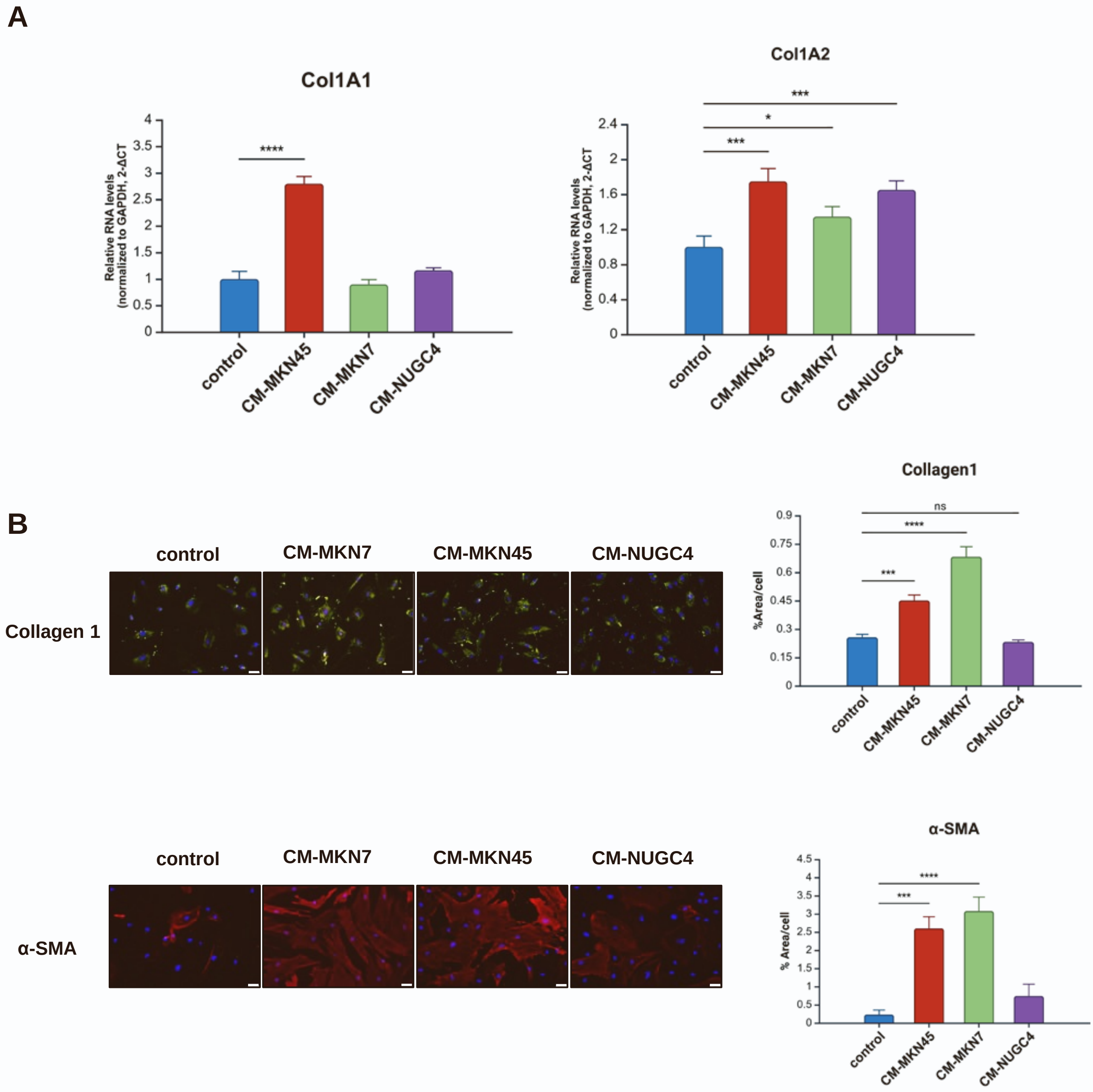

Fig. S2
The functional changes of FEF3 cells after incubation with conditioned medium of gastric cancer cells. **A)** Expression of Col1A1 and Col1A2 mRNA in FEF3 cell after incubation with condition medium (CM) of each human gastric cancer cells for 4 days. Cells were analyzed using quantitative RT-PCR analysis. Data are expressed as mean ± SD (n=3). **B)** Representative images of immunocytochemical staining of collagen1 and α-SMA in FEF3 after incubation with CM of each GC cells for 4 days. Serum-free medium (SFM) was used as a control. The area index for each staining was evaluated by Image J software. Data are expressed as mean ± SD (n=3). Scale bar, 50 μm. *$p<0.05$, ***$p<0.001$, ****$p<0.0001$.

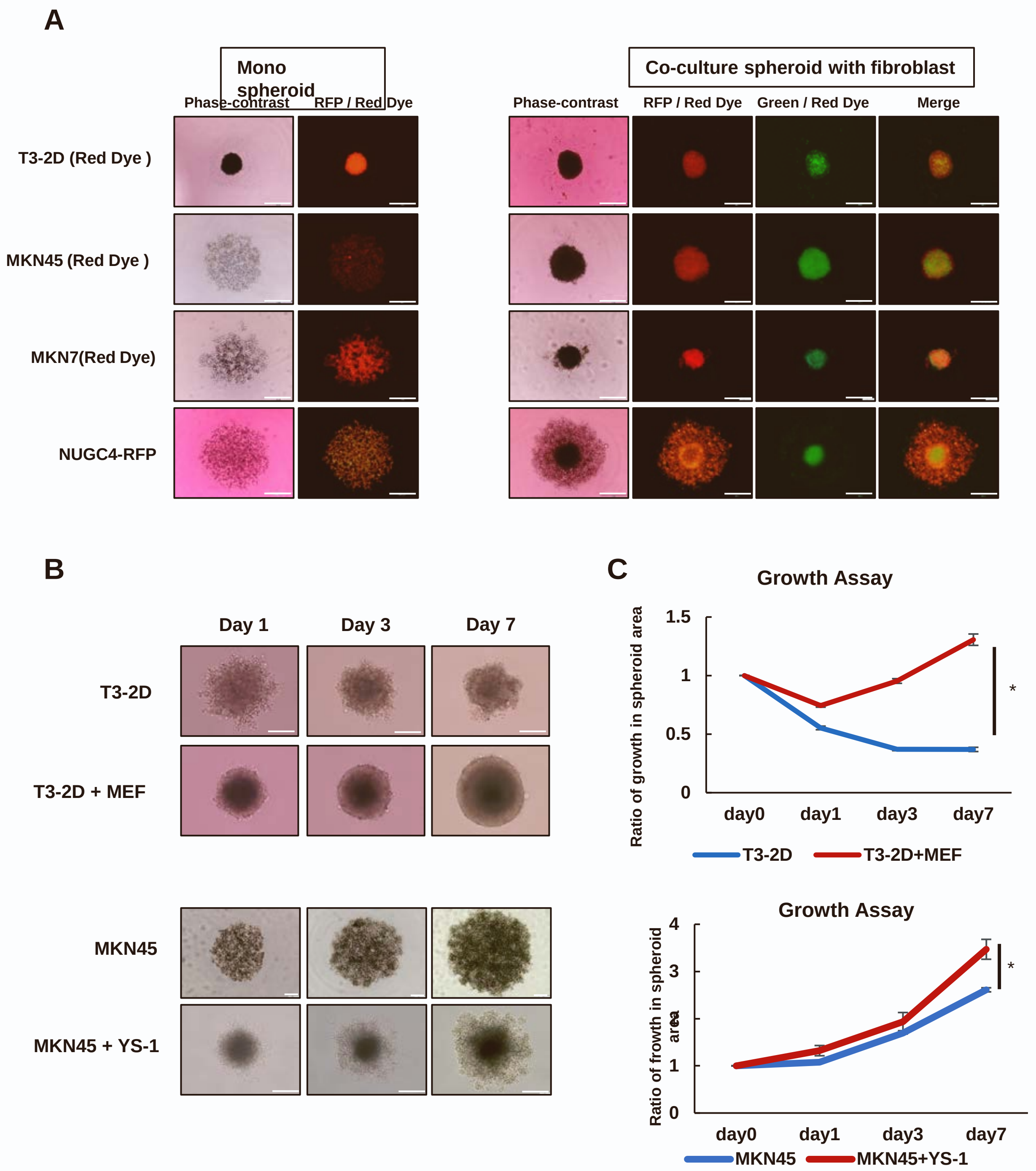

Fig. S3

Representative microscopic images of spheroids of gastric cancer cells and co-cultured with fibroblasts. **A)** Mono spheroids were cultured with 5000 gastric cancer (GC) cells and co-cultured spheroids were cultured with 5,000 GC cells and 10,000 fibroblasts for 48 hours. GC cells were stained with 10 mM CellTrackerTM Red CMTPX Dye and fibroblasts were stained with 10 mM CellTrackerTM Green CMFDA Dye. Scale bar, 200 µm. **B)** Representative microscopic images of growth of mono-spheroids or co-cultured spheroids over time for 7 days. Scale bar, 100 µm. **C)** Data are expressed as ratio of change in spheroid circumference ± SD (n = 5). Statistical significance was determined using Liner Regression. *, $P < 0.05$.

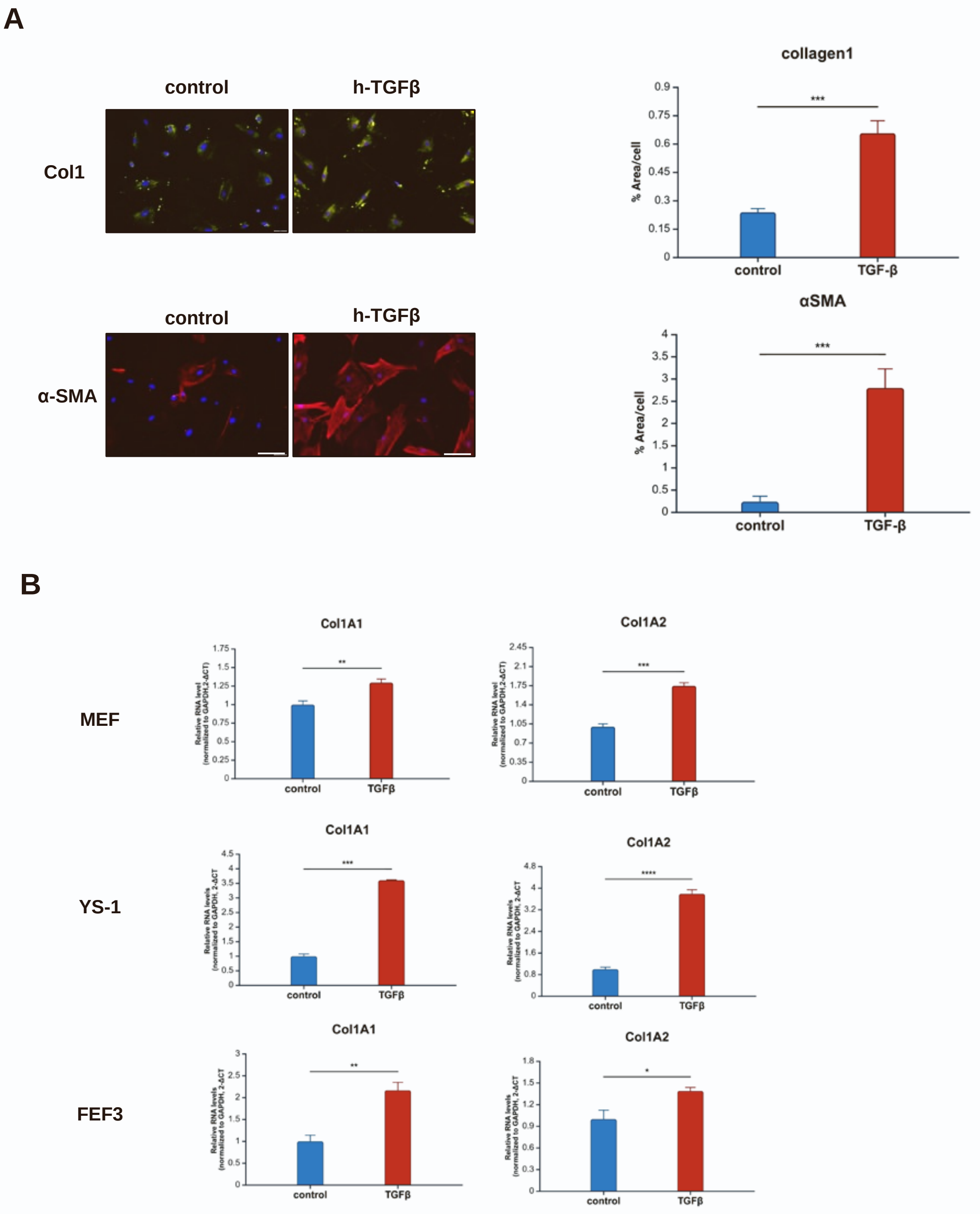

Fig. S4

The functional changes of fibroblasts after incubation with TGF-β. **A)** Representative images of immunocytochemical staining of collagen1 and α-SMA in FEF3 cell after incubation with recombinant TGF-β (10 ng/ml) for 4 days. SFM was used as a control. The area index for each staining was evaluated by Image J software. Data are expressed as mean ± SD (n=3). Scale bar, 100 μm. **B)** Expression of Col1A1 and Col1A2 mRNA in MEF, YS-1 and FEF3 cells after incubation with recombinant TGF-β (10 ng/ml or 100 ng/ml) for 4 days. Cells were analyzed using quantitative RT-PCR analysis. Data are expressed as mean ± SD (n=3). *$p<0.05$, **$p<0.01$, ***$p<0.001$, ****$p<0.0001$.

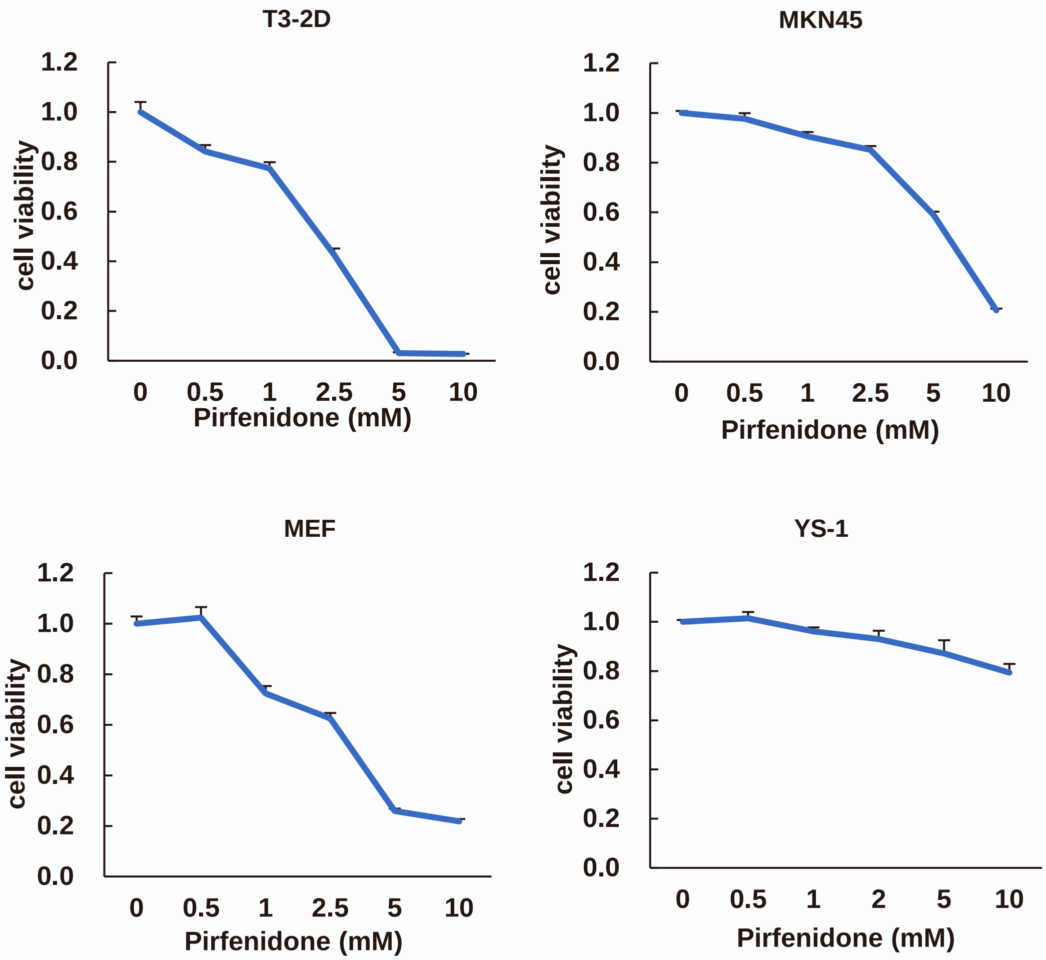

Fig. S5
Cell viability assay of gastric cancer cells and fibroblasts after treated with Pirfenidone. T3-2D, MKN45, MEF and YS-1 cells were treated with Pirfenidone at the concentration of 0, 0.5, 1, 2.5, 5 or 10 mM for 72 hours. Cell viability assessed using the XTT assays. Cell viability was calculated relative to that of the mock-treated cells, which were set as 1.0. Data are expressed as mean ± SD (n = 5).

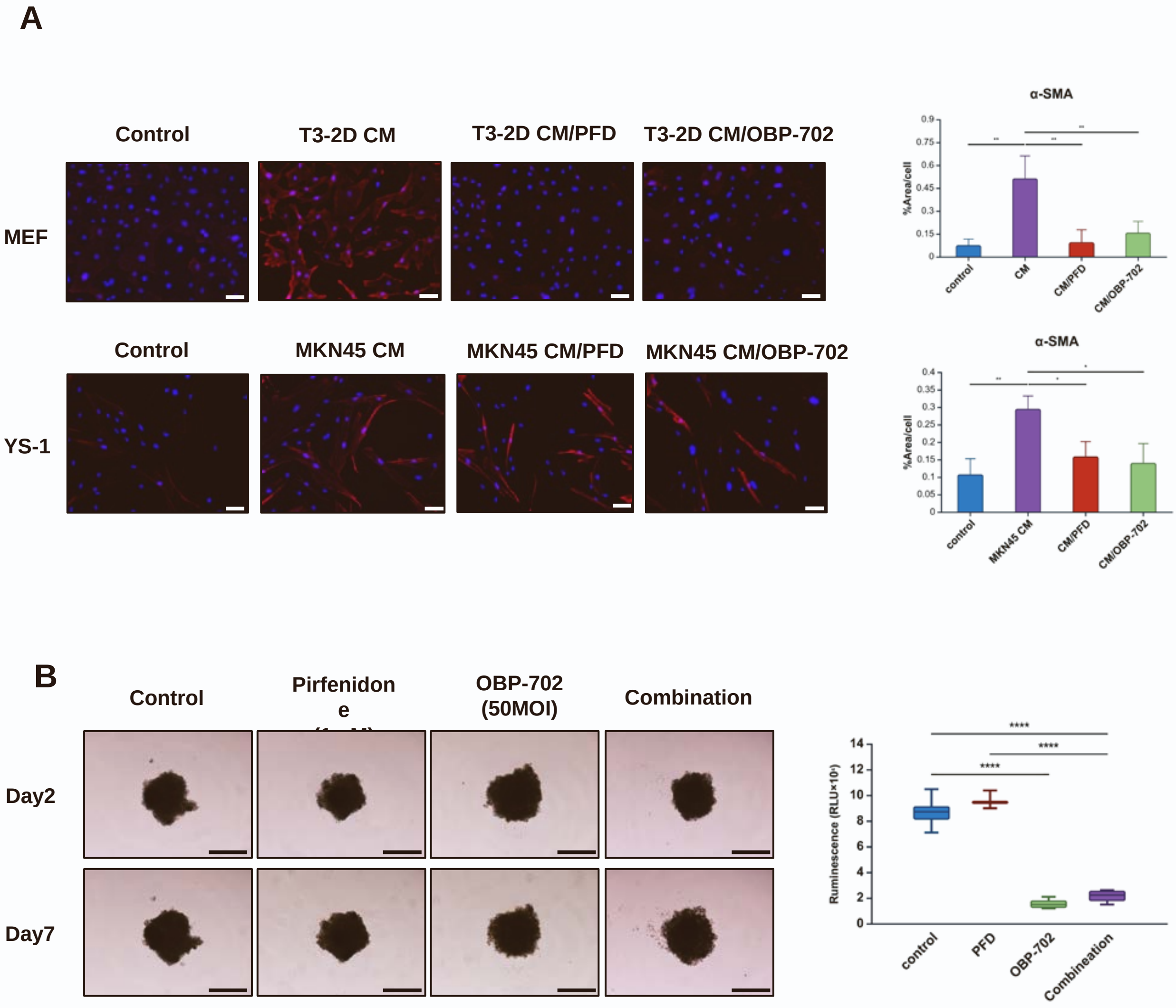

Fig. S6

The efficacy of Pirfenidone and OBP-702 on CAFs and spheroids od cancer cells and fibroblasts. **A)** Representative images of immunocytochemical staining of α-SMA in MEF and YS-1 cells after incubation with CM of T3-2D or MKN45 cells and treated with Pirfenidone (PFD) (1mM) or OBP-702 (20MOI or 50MOI) for 4 days. SFM was used as a control. The area index for each staining was evaluated by Image J software. Data are expressed as mean ± SD (n=3). Scale bar, 100 μm. **B)** Representative images of MKN45 co-cultured with YS-1 cells spheroids after treated with PFD (1mM), OBP-702 (50MOI) or the combination for 7 days. ATP cell viability assay of co-culture spheroid after treated with PFD (1mM), OBP-702 (50MOI) or the combination at 7 days after treatment. Data are expressed as mean ± SD (n = 5). *p<0.05, **p<0.01, ****p<0.0001.

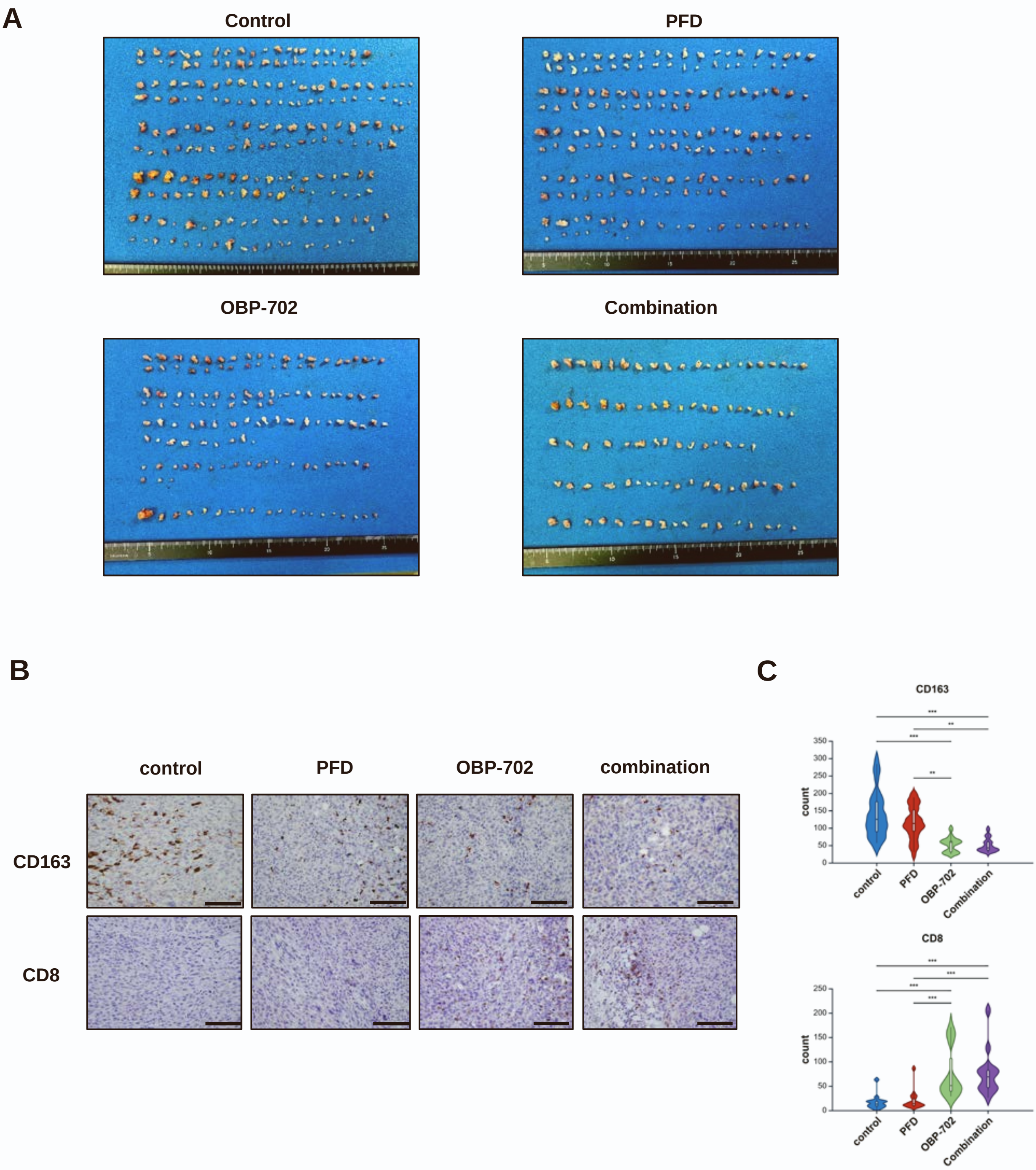

Fig. S7
Anti-tumor effects of Pirfenidone and OBP-702 on orthotopic mouse peritoneal metastasis model of T3-2D and co-injected with MEF. **A)** Macroscopic images of peritoneal nodules of in each group. **B)** Representative microscopic images of CD8 and CD163 staining of peritoneal nodules in each group. Scale bar, 100 µm. **C)** The mean cell numbers of CD8 and CD163 expression in peritoneal tumors treated with each treatment. The mean cell numbers were calculated from three selected fields in each mouse, from a total of five mice per group. Data are expressed as the mean ± SD (n = 5). *p<0.05, **p<0.01, ***p<0.001, ****p<0.0001.

Table. S1

Patients' demographics and pathological characteristics of 106 advanced gastric cancer patients.

		Collagen high (n=53)	Collagen low (n=53)	p-value
**Background**	Age (median, years)	65	74	<0.001
	Sex; M: F	40: 13	37: 16	n.s
	BMI (median)	22.3 (20.7-24.0)	22.3 (19.5-25.6)	n.s
**Pathological diagnoses**	SS: SE	20: 33	25: 28	n.s
	pStage[a]; II: III: IV	20: 24: 9	28: 18: 7	n.s
	Histological type; Intestinal: diffuse	14: 39	29: 24	0.0053
	ly; 0 or 1: 2 or 3	6: 47	17: 36	0.017
	v;0 or 1: 2 or 3	17: 36	19: 34	n.s
	Lymph node metastasis	45	35	0.041
	αSMA (median)	2.601 (1.93-3.65)	2.09 (1.54-2.61)	0.0026
**Recurrence**	All	32	15	0.0016
	Peritoneal metastasis	23	8	0.0025
	Liver metastasis	5	7	n.s

[a]Tumor stage is classified by Japanese Classification of Gastric Carcinoma, 3rd English edition

Table. S2

Primer sequences for qRT-PCR.

**Primers for qRT-PCR**	
**Gene**	**Primer sequence**
GAPDH (human)	Forward: CTGCACCACCAACTGCTTAG<br>Reverse: GTCTTCTGGGTGGCAATGAT
Col1A1 (human)	Forward: GATTCCCTGGACCTAAAGGTGC<br>Reverse: AGCCTCTCCATCTTTGCCAGCA
Col1A2 (human)	Forward: CCTGGTGCTAAAGGAGAAAGAGG<br>Reverse: ATCACCACGACTTCCAGCAGGA
ACTA2 (human)	Forward: CTATGCCTCTGGACGCACAACT<br>Reverse: CAGATCCAGACGCATGATGGCA
