# Supplementary material for: Collagen depletion by pirfenidone enhances antitumor effect of oncolytic adenovirus against peritoneal metastases of gastric cancer
Source: Mol Ther Oncol. 2025 Sep 2;33(4):201045. doi: 10.1016/j.omton.2025.201045 (PMC12859411; doi:10.1016/j.omton.2025.201045)
Supplement: Document S2. Article plus supplemental information [file mmc2.pdf]

Molecular Therapy
Oncology
Original Article

Collagen depletion by pirfenidone enhances antitumor effect of oncolytic adenovirus against peritoneal metastases of gastric cancer

Tomohiro Okura,[1] Satoru Kikuchi,[1] Hiroshi Tazawa,[1,2] Yu Mikane,[1] Nobuhiko Kanaya,[1] Ema Mitsui,[1] Yuta Une,[1] Kunitoshi Shigeyasu,[1] Toshiaki Ohara,[1,3] Shinji Kuroda,[1] Kazuhiro Noma,[1] Junko Ohtsuka,[4] Rieko Ohki,[4] Shunsuke Kagawa,[1] Yasuo Urata,[5] and Toshiyoshi Fujiwara[1]

[1]Department of Gastroenterological Surgery, Okayama University Graduate School of Medicine, Dentistry and Pharmaceutical Sciences, Okayama 700-8558, Japan; [2]Center for Innovative Clinical Medicine, Okayama University Hospital, Okayama 700-8558, Japan; [3]Department of Pathology and Experimental Medicine, Okayama University Graduate School of Medicine, Dentistry and Pharmaceutical Sciences, Okayama 700-8558, Japan; [4]Laboratory of Fundamental Oncology, National Cancer Center Research Institute, Tokyo 104-0045, Japan; [5]Oncolys BioPharma, Inc., Tokyo 106-0032, Japan

Cancer-associated fibroblasts (CAFs) play a crucial role in collagen accumulation, which develops and promotes peritoneal metastasis (PM) in gastric cancer (GC). In addition, the abundant stromal collagens in the tumor microenvironment function as a physical barrier against penetration of antitumor drugs and oncolytic viruses. This study investigated whether collagen depletion by pirfenidone (PFD), an antifibrotic drug, enhances the antitumor effects of oncolytic adenoviruses. Analysis of the clinical samples revealed a significant association of high expression of collagen 1 and α-smooth muscle actin (α-SMA) with PM development and poor prognosis of advanced GC. Human and murine GC cells enhanced collagen production by fibroblasts, which was suppressed by PFD. Abundant fibroblasts and collagen inhibited the penetration of OBP-702, which reduced the antitumor effects of OBP-702 in the spheroid model. Intraperitoneal co-injection of GC cells and fibroblasts promoted the development of collagen-rich PM and reduced the antitumor effects of OBP-702 *in vivo* model. PFD suppressed collagen production in PM and improved viral penetration into the tumors, which enhanced the antitumor effects of OBP-702 against PM of GC. Collagen depletion by PFD enhances the penetration of OBP-702 into PM of GC, in turn enhancing the antitumor effects of OBP-702 against PM of GC.

INTRODUCTION

There were over one million new cases of gastric cancer (GC) in 2020. Ranking fifth for incidence and fourth for mortality, GC remains an important cancer worldwide.[1] GC is generally divided into two major sub-types based on histological characteristics: intestinal and diffuse. Intestinal-type GC (IGC) has a better prognosis than diffuse-type GC (DGC), with metastases predominantly to the liver, whereas peritoneal metastases are more likely in DGC.[2] In DGC, alterations in the tumor microenvironment (TME) also contribute to invasion and metastasis, in addition to genomic alterations. Compared to early stage DGC and IGC, as well as healthy gastric tissue, advanced DGC shows greater accumulation of extracellular matrix (ECM) components.[3–5] Cancer-associated fibroblasts (CAFs) are much more abundant in DGC than IGC tumors, and tumor infiltration of CAFs correlates with poor survival of DGC patients. The malignant behavior of DGC tumors is highly influenced by interaction with CAFs and ECM by paracrine signaling, such as by TGF-beta and by ECM organization.[6,7] Collagen is one of the major proteins in the ECM. During tumor progression, CAFs perform important roles that lead to tumor fibrosis through dysregulated collagen turnover.[8] Although the relationship between GC and collagen is still not fully understood, it has been suggested that the gene for collagen 1 could be a prognostic factor in GC, and that the collagen signature of the serosal invasion could be a predictor of peritoneal metastasis (PM) after surgery.[9,10] Furthermore, upregulation of collagen-related genes in GC is related to poor prognosis and might indicate poor responsiveness to immunotherapy.[11] Therefore, collagen itself might be a therapeutic target in GC; however, effective collagen-targeted therapies for GC have not yet been established.

Oncolytic virotherapy (OV) is a recently developed treatment modality that is expected to have antitumor effects against PM of GC.[12] We have previously developed a telomerase-specific replication-competent oncolytic adenovirus, OBP-301 (suratadenoturev), which drives the E1A and E1B genes for viral replication under the control of human telomerase reverse transcriptase promoter, and have confirmed its antitumor effects in various human tumor cells.[13–15] We have shown that intraperitoneal administration of

Received 5 June 2025; accepted 27 August 2025;
https://doi.org/10.1016/j.omton.2025.201045.

Correspondence: Satoru Kikuchi, Department of Gastroenterological Surgery, Okayama University Graduate School of Medicine, Dentistry and Pharmaceutical Sciences, Okayama 700-8558, Japan.
E-mail: satorukc@okayama-u.ac.jp

© 2025 The Author(s).
Published by Elsevier Inc. on behalf of The American Society of Gene and Cell Therapy.
This is an open access article under the CC BY license (http://creativecommons.org/licenses/by/4.0/).

OBP-301 synergistically suppressed PM of GC in combination with paclitaxel.[16] A phase I study conducted in the United States has already confirmed the safety and biological activity of intra-tumoral administration of OBP-301 in patients with several types of solid tumor.[17] To promote the antitumor effects of OBP-301, we have developed OBP-702 as a modification of OBP-301 that expresses the wild-type p53 gene. OBP-702 exhibits greater antitumor effects than OBP-301 through the induction of apoptosis and oncolysis in various types of tumor cells via exogenous p53 overexpression in tumor cells.[18,19] We have shown that intraperitoneal administration of OBP-702 significantly suppressed the metastatic tumor formation of both p53-intact and p53-mutant GC cells, and combination therapy with OBP-702 and paclitaxel significantly suppressed PM compared with OBP-702 monotherapy.[20,21] Furthermore, OV immunotherapy is considered a highly promising approach for treating patients with various cancers.[22] We have recently shown that intraperitoneal administration of OBP-702 restores antitumor immunity via the remodeling of tumor-associated macrophages (TAMs) in addition to direct tumor lysis, and cooperates with immune checkpoint inhibitors (ICIs) to suppress PM of GC.[23] However, highly abundant ECM can serve as a barrier that inhibits penetration of OV into the tumors. It has been shown that viruses engineered to carry enzymes such as hyaluronidase can modify ECM and improve viral spreading.[24] Therefore, combination therapy with drugs that modify ECM may improve viral penetration and enhance the antitumor effects of OV.

Pirfenidone (PFD) is approved for the management of idiopathic pulmonary fibrosis (IPF) as an antifibrotic drug. PFD is an inhibitor of TGF-β production and TGF-β stimulated collagen production and also has antifibrotic properties.[25] A recent study has reported targeting fibrosis through TGF-β signaling as a cancer therapy,[26] and PFD has been the focus of other studies that have evaluated its antitumor effects, targeting CAFs in malignant tumors. There are several reports of therapeutic strategies that have applied the antifibrotic effect of PFD against pancreatic and colorectal cancers.[27–29] However, effects of the combination of OV and PFD have not yet been reported.

In the present study, higher expression of collagen was confirmed in immunohistochemical (IHC) analysis of clinical samples of advanced GC as well as PM of GC. We investigated the effects of collagen and CAFs on the therapeutic efficacy of OBP-702 in *in vitro* and *in vivo* PM mouse models. Furthermore, we investigated the therapeutic potential of PFD for enhancing the antitumor effects of OBP-702 against PM of GC.

RESULTS

Higher collagen expression in advanced GC correlates with poor prognosis and PM

To investigate whether collagen expression levels in advanced GC correlate with prognosis, we evaluated collagen and α-SMA (as a marker of CAFs) in clinical samples from 106 cases of advanced GC using IHC staining analysis (Figure 1A). The calculated median area indexes were 17.97% for collagen and 2.25% for α-SMA, and the cases were divided into two groups based on these median values (Figure 1B). A positive correlation was observed between the expression levels of collagen and α-SMA, with a particularly strong positive correlation shown in DGC (Figure 1C). Patients with higher collagen expression or higher α-SMA expression had significantly shorter OS and RFS than those with lower expression (Figures 1D and S1A). Database analysis using the Kaplan-Meier plotter also showed that for stage III or IV GC cases, patients with the higher RNA expression of Col1A1 or Col1A2 showed significantly shorter OS (Figure S1B).

In examination of the expression of collagen and α-SMA in PM of GC, IHC staining was performed in the same manner in 20 cases of PM and in 8 cases of normal peritoneum. Cases of PM showed significantly higher expression of collagen and α-SMA compared to non-PM (Figure 1E). Positive correlation was also observed between the expression levels of collagen and α-SMA in PM of GC (Figure 1F). These results suggest that collagen in advanced GC is associated with poor prognosis and might be involved in the development of PM of GC.

GC cells increase collagen 1 expression in fibroblasts and collagen increases proliferation of GC cells

To assess the functions of collagen 1 expression, both Col1a1 and Col1a2 chains (encoded by the Col1A1 and Col1A2 genes, respectively) were detected. These chains are components of the functional unit of collagen 1. Therefore, the mRNA levels of Col1A1, Col1A2, and ACTA2 in murine and human GC cell lines and fibroblast cell lines were measured by RT-PCR analysis. The expression levels of these collagen-related genes were lower in both murine and human GC cell lines compared to fibroblasts (Figure 2A). The mRNA expression levels of Col1A1 and Col1A2 increased significantly after incubation of fibroblasts with GC-CM (Figures 2B and S2A). Immunofluorescence staining also showed that the intracellular expression levels of collagen 1 and α-SMA were significantly increased with GC-CM (Figures 2C and S2B). These results suggest that both DGC and IGC cell lines induce collagen 1 expression in fibroblasts. We then investigated whether collagen increases the proliferation of cancer cells. Proliferation of T3-2D and MKN45 cells was significantly increased after stimulation with 10% collagen for 72 h, compared to the unstimulated condition (Figure 2D). Aggregation and proliferation of T3-2D and MKN45 cells were increased after 10% collagen stimulation compared to the unstimulated condition (Figure 2E). Direct interaction between GC cells and fibroblasts was evaluated using 3D co-culture spheroid models. GC cells formed spheroids with a core in the center when co-cultured with fibroblasts (Figure S3A). Co-culture spheroids of T3-2D and MEF, and of MKN45 and YS-1, formed larger spheroids over time compared to the monoculture spheroids (Figures S3B and S3C). These results suggest that collagen 1 increases the proliferation of GC cells, and that spheroid formation is enhanced through direct interaction with fibroblasts.

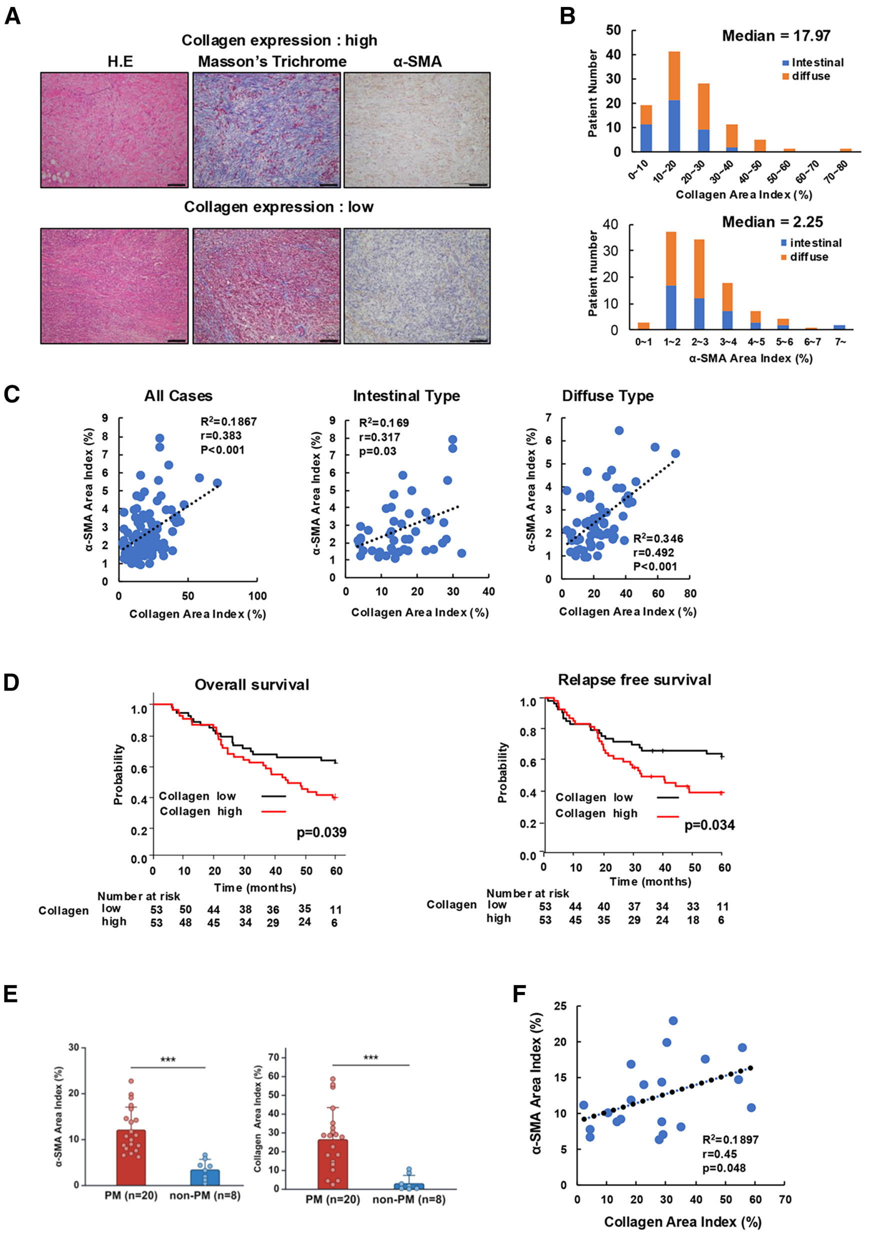

Figure 1. Analysis of collagen and α-SMA expression in clinical samples of advanced gastric cancer and peritoneal metastasis

(A) Representative microscopic images with H&E, Masson trichrome, and α-smooth muscle actin (α-SMA) staining of high or low collagen expression in advanced gastric cancer (GC). Scale bars, 100 μm. (B) The area index for each staining was evaluated by ImageJ software. The area indexes of collagen and α-SMA for all patients are plotted as histograms. (C) Correlation between collagen and α-SMA expression is shown in scatterplots (Spearman's correlation coefficient) for all advanced GC, intestinal, and diffuse-type cases. (D) Overall survival and relapse-free survival curves of advanced GC patients according to collagen expression (high or low) in the tumor. The high-collagen expression group showed significantly worse prognosis (log rank test). (E) Comparison in collagen and α-SMA expression between peritoneal metastasis (PM) (20 cases) and non-PM (8 cases). The area index for each staining was evaluated by ImageJ software. (F) Correlation between collagen and α-SMA expression is shown in a scatterplot (Spearman's correlation coefficient) of PM cases.

Collagen and fibroblasts inhibit oncolytic virus penetration and reduce antitumor effects

To evaluate the antitumor effects and toxicity of OBP-702 against human and murine GC cells and fibroblasts, T3-2D, MKN45, MEF, and YS-1 cells were treated with OBP-702. Cytotoxic effects were confirmed in all GC cells in a dose-dependent manner after infection with OBP-702, whereas moderate cytotoxicity of OBP-702 was confirmed in fibroblasts at high doses (Figure 3A). The antitumor effects of OBP-702 were reduced after stimulation in CM containing 10% collagen (Figure 3B). T3-2D and MKN45 cells cultured with 10% collagen-supplemented medium formed colonies (Figure 3C). In investigation of the effect of collagen on the antitumor effect of OBP-702 in 3D spheroid culture, monoculture spheroids of GC cells in medium containing 2% collagen exhibited significantly higher ATP activity after treatment with OBP-702 for 7 days compared to spheroids without collagen medium (Figures 3D and 3E). OBP-401 is an Ad variant of OBP-301 that enables monitoring of viral replication in cancer cells via GFP expression. In the spheroids co-cultured with fibroblasts, OBP-401 could not penetrate the central part of the spheroids (Figure 3F). These results suggest that collagens and fibroblasts may physically inhibit the penetration of OBP-702 into the tumor.

Antitumor effect of OBP-702 was attenuated in collagen-rich PM of GC

To investigate the influence of collagen and fibroblasts in PM on intraperitoneal OBP-702 therapy, we inoculated cancer cells (T3-2D) and T3-2D co-cultured with fibroblasts (MEF) into the peritoneal cavity of C57BL/6J mice. Four intraperitoneal administrations of PBS or OBP-702 were performed in total, and peritoneal metastasis tissues were collected at 17 days after tumor inoculation (Figures 4A and 4B). The total number of tumors and the tumor weight in the co-inoculated group were significantly higher than those in the T3-2D alone group. Regarding therapeutic effect, the antitumor effect of OBP-702 was significantly attenuated in the co-inoculated T3-2D and MEF group compared to the T3-2D alone group (Figures 4C and 4D). Masson's trichrome staining and IHC staining of collagen 1 revealed significantly higher stromal expression in the co-inoculated T3-2D and MEF group

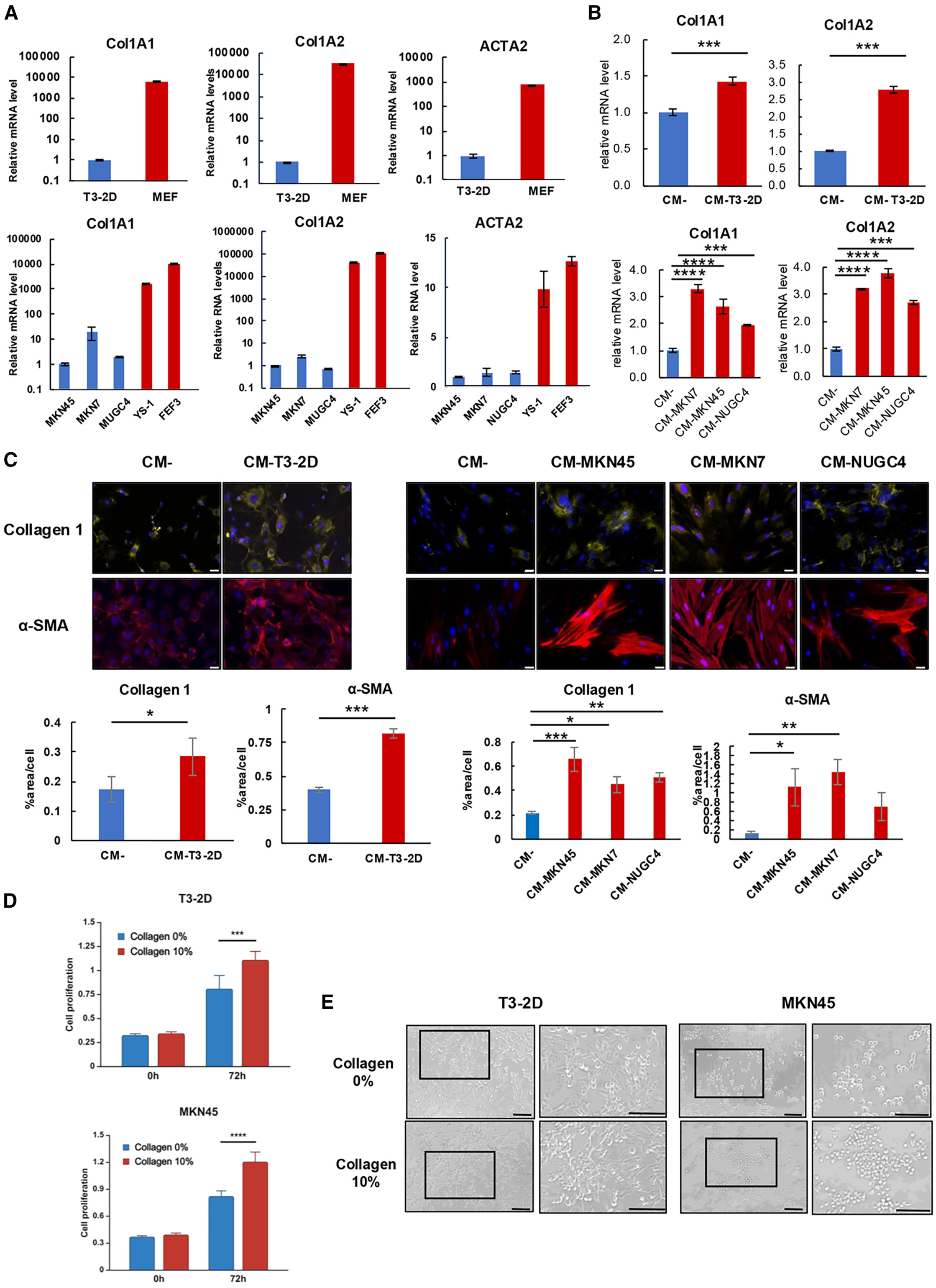

Figure 2. GC cells increase collagen 1 expression in fibroblasts and collagen increases proliferation of GC cells

(A) Expressions of Col1A1, Col1A2, and ACTA2 mRNA in mouse gastric cancer (GC) cells (T3-2D), human GC cells (MKN45, MKN7, and NUGC4), mouse fibroblast cells (MEF), and human fibroblast cells (YS-1 and FEF3) are shown. All cells were analyzed by quantitative RT-PCR analysis. Data are expressed as the mean ± SD (n = 3).

(legend continued on next page)

compared to the T3-2D alone group (Figures 4E and 4F). These results suggest that collagen within the tumor is involved in attenuation of the antitumor effects of OBP-702 in addition to enhancing tumorigenesis.

PFD inhibits collagen expression in fibroblasts and increases the antitumor effect of OBP-702 on spheroid models

It is known that stimulation of TGF-β1 induces the expression of collagen in fibroblasts. Analysis of TGF-β1 levels in each GC-CM using ELISA revealed elevated TGF-β1 concentration in CM of the T3-2D, MKN45, MKN7, and NUGC4 cells (Figure 5A). Immunofluorescence staining confirmed that the expressions of collagen 1 and α-SMA were upregulated in MEF, YS-1, and FEF3 by stimulation with TGF-β1 (Figure 5B and S4A). The mRNA expression levels of Col1A1 and Col1A2 in MEF, YS-1, and FEF3 were significantly higher in fibroblasts stimulated with TGF-β1 than in unstimulated fibroblasts (Figure S4B). Investigation of the combined effects of PFD and OBP-702 found that PFD reduced cell viability of GC cells in a dose-dependent manner, whereas it had slight cytotoxicity to fibroblasts at high doses (Figure S5). The expression of α-SMA in MEF and YS-1 cells increased when treated with GC-CM, and these upregulated expressions were decreased by both PFD and OBP-702 (Figure S6A). However, the expression of collagen 1 was suppressed by PFD but not by OBP-702 (Figure 5C). To investigate the effect of PFD on viral penetration, spheroids of GC cells co-cultured with fibroblasts were treated with PFD and OBP-702, and ATP assay was performed on day 7. The group treated with the combination of PFD and OBP-702 showed significant inhibition of spheroid growth as well as the lowest ATP activity compared to the other groups (Figures 5D, 5E, S6B, and S6C). Penetration of OBP-702 into the spheroids was evaluated by IHC staining. In spheroids of T3-2D co-cultured with MEF, collagen accumulated inside the spheroids, and OBP-702 monotherapy could not penetrate the interior. PFD decreased collagen accumulation in the spheroids, which enabled OBP-702 to penetrate the interior (Figure 5F).

Collagen depletion by PFD enhances the antitumor effects of OBP-702 in peritoneal metastasis

We combined PFD with OBP-702 to confirm whether PFD could enhance the antitumor effects of intraperitoneal OBP-702 treatment by collagen depletion. Intraperitoneal OBP-702 and/or PFD treatment was performed for orthotopic mouse PM model established from T3-2D cells co-inoculated with MEF (Figure 6A). PM formation was significantly suppressed in the combined PFD and OBP-702 treatment group compared to the PFD and OBP-702 monotherapy groups (Figures 6B and S7A). In mice bearing PM, OS was significantly prolonged in the combination therapy group than in the other groups (Figure 6C). Masson's trichrome staining and IHC of collagen 1 and α-SMA revealed that collagen expression within the PM tumor was significantly decreased in the combination treatment group (Figures 6D and 6E). Furthermore, IHC staining analysis demonstrated significantly higher detection of adenovirus V proteins in the combination treatment group than in the OBP-702 monotherapy groups (Figure 6E). These results suggest that PFD may reduce collagen accumulation, and thus facilitate penetration of OBP-702 into PM tumors. Moreover, IHC staining analysis showed that combination therapy significantly increased the number of $CD8^+$ tumor-infiltrating lymphocytes (TILs) and significantly decreased the number of $CD163^+$ cells compared with PFD monotherapy (Figures S7B and S7C).

DISCUSSION

Peritoneal metastases exhibit a distinctive pattern of metastasis and recurrence in GC and are associated with poor prognosis as they are refractory to conventional treatment.[2] CAFs have been proven to play an important role in tumorigenesis and progression of tumors and also in the development of PM in GC.[21,30] In addition, CAFs are one of the most important cell types in remodeling of ECM components such as collagen, which contributes to tumor growth and drug resistance.[8,31] OV is a promising intraperitoneal therapeutic modality for PM, in addition to intraperitoneal chemotherapy. The present study showed that intraperitoneal administration of OBP-702 synergistically suppressed PM in combination with an antifibrotic agent that causes collagen depletion in the tumors.

Collagen is one of the main components of the ECM, and its remodeling is involved in the development and progression of various cancers.[32] Recently, collagen accumulation in solid tumors has been implicated in immunosuppression, and is expected to be a novel therapeutic target.[11,33] Previous studies have shown that PM of GC is caused by serosal invasion of the primary tumor, and that the collagen signature in the serosal invasion site could be a predictor of PM recurrence.[10,34] Furthermore, it has been reported that CAFs promote PM in ovarian cancer through the production of collagen.[35] Therefore, we considered that collagen-targeted therapy could be a promising treatment strategy for PM of GC. The present finding that collagen was highly expressed in primary advanced GC and PM tumors suggests that the collagen expression level of primary GC may be a poor prognostic factor for advanced GC and could be associated with the development of PM. Moreover, collagen stimulation promoted proliferation of the mouse gastric cancer cell line, T3-2D; and of the human DGC cell line, MKN45. These cancer cells were observed to aggregate and proliferate, using collagen as a

(B) Expression of Col1A1 and Col1A2 mRNA in MEF (upper) and YS-1 (lower) cells after incubation with serum-free medium (SFM) as a control and conditioned medium (CM) of each GC cell type for 4 days. Cells were analyzed using quantitative RT-PCR analysis. Data are expressed as the mean ± SD (n = 3). (C) Representative images of immunocytochemical staining of collagen 1 and α-SMA in MEF (left) and YS-1 (right) after incubation with normal medium and CM of each of the GC cells for 4 days. SFM was used as a control. The area index for each staining was evaluated by ImageJ software. Data are expressed as the mean ± SD (n = 3). Scale bars, 50 μm. (D) Cell proliferation of T3-2D and MKN45 cells with and without 10% collagen stimulation for 72 h. Data are expressed as the mean ± SD (n = 5). (E) Representative cellular morphological images of T3-2D and MKN45 cells with and without 10% collagen 1 stimulation for 72 h. Scale bar, 100 μm. *p < 0.05, **p < 0.01, ***p < 0.001, ****p < 0.0001.

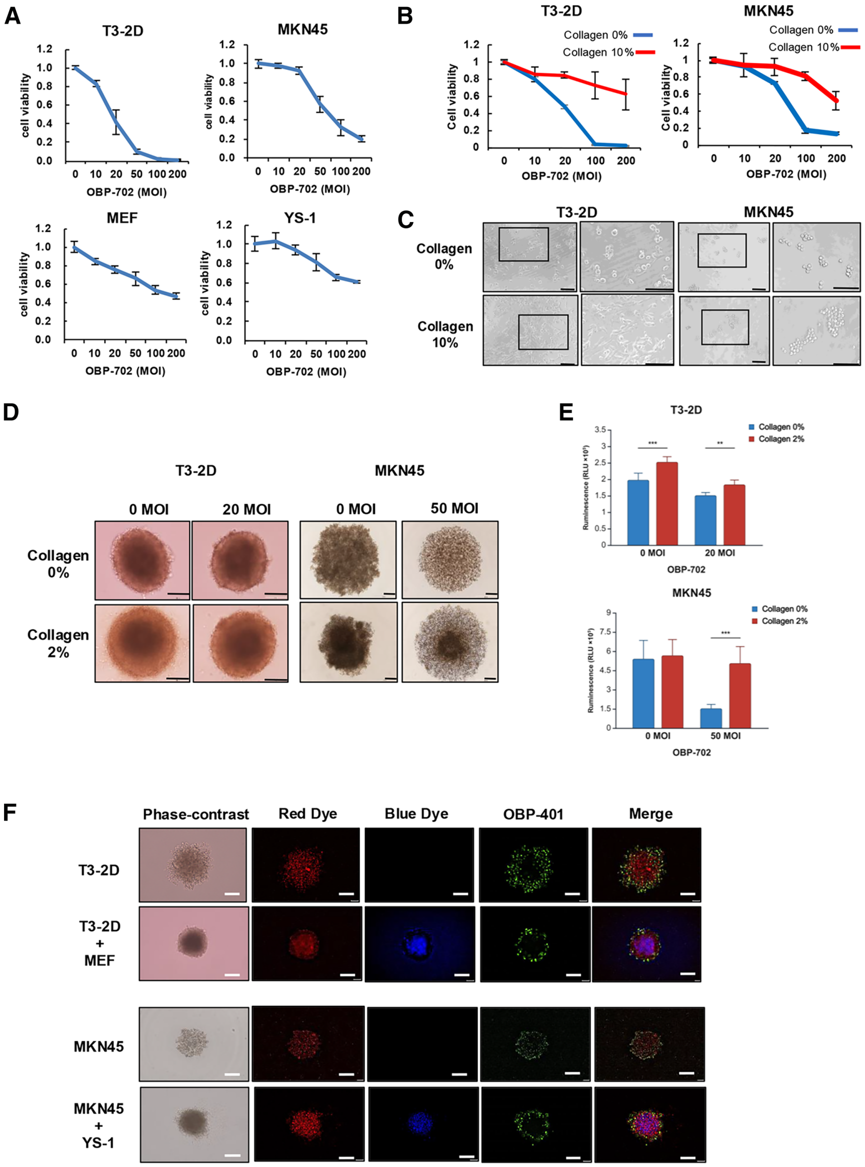

Figure 3. Collagens and fibroblasts inhibit oncolytic virus penetration and reduce antitumor effects

(A) T3-2D, MKN45, MEF, and YS-1 cells were infected with OBP-702 at the indicated MOIs for 3 days. Cell viability was assessed using the XTT assays. Cell viability was calculated relative to that of the mock-infected cells, which were set as 1.0. Data are expressed as the mean ± SD (n = 5). (B) T3-2D and MKN45 cells were infected with OBP-702 at the indicated MOIs for 3 days with or without 10% collagen containing medium. Cell viability was assessed using the XTT assays. Cell viability was calculated relative to that of the mock-infected cells, which were set as 1.0. Data are expressed as the mean ± SD (n = 5). (C) Representative cellular morphological images of T3-2D and MKN45 cells infected with 100 MOI of OBP-702 with or without 10% collagen stimulation for 72 h. Scale bars, 100 μm. (D) Representative spheroid morphological images of T3-2D cells infected with 20 MOI of OBP-702 or MKN45 cells infected with 50 MOI of OBP-702 with or without 2% collagen stimulation for 7 days. Scale bars, 100 μm. (E) ATP cell viability assay of T3-2D and MKN45 spheroids with or without 2% collagen stimulation for 7 days after infection with OBP-702 (20 MOI or 50 MOI). Data are expressed as the mean ± SD (n = 5). (F) Representative images of mono-spheroids and co-cultured spheroids infected with OBP-401 (100 MOI) for 48 h. T3-2D and MKN45 cells were labeled with red cell trackers and MEF and YS-1 cells with blue cell trackers. Scale bars, 50 μm. $^{**}p < 0.01$, $^{***}p < 0.001$.

scaffold. Furthermore, it has been reported that collagen promotes the proliferation and invasion of cancer cells via the FAK-AKT signaling pathway.[36,37] These functions of collagen are known to be associated with the proliferation and invasion of cancer cells.

A previous study has reported that collagen fibers inhibited the penetration of OV into the tumors, and that dissolving collagen with collagenase improved the penetration of OV.[38] The present study showed that the presence of collagen in the CM reduced the antitumor effect of OBP-702. Together, these results suggest that the presence of collagen inhibits the penetration of OBP-702 and also increases cancer cell proliferation. In the 3D co-culture spheroid model of cancer cells and fibroblasts, collagen accumulation was observed within the spheroid, and OBP-702 did not penetrate the interior of the spheroid. Since the clinical use of collagenase for cancer patients is not currently available, it is necessary to consider the use of alternative available antifibrotic drugs. *Chen Y* et al. reported that antifibrotic therapy might enhance the antitumor effects of oncolytic vesicular stomatitis virus.[39] It has been reported that several OV encoding the expression of ECM degrading agents including hyaluronidase, decorin, and relaxin enhanced the viral penetration and distribution into the tumor, resulting in increasing the anti-tumor effects of OV. Moreover, ECM remodeling by these OVs also enhanced the delivery of chemotherapeutic agents and several therapeutic antibodies and increased the sensitivity of ICIs.[40–43] However, the expression of these ECM degrading agents depends on the replication and distribution of OVs themselves. Therefore, the antifibrotic agent PFD could be expected to enhance the therapeutic effect of OBP-702 regardless of the replication and distribution of OV itself.

PFD has already been applied clinically for the treatment of IPF and has shown antifibrotic efficacy in the treatment of several cancers.[25,27–29] With regard to its antitumor effects by remodeling ECM in malignant tumors, it has been reported to inhibit

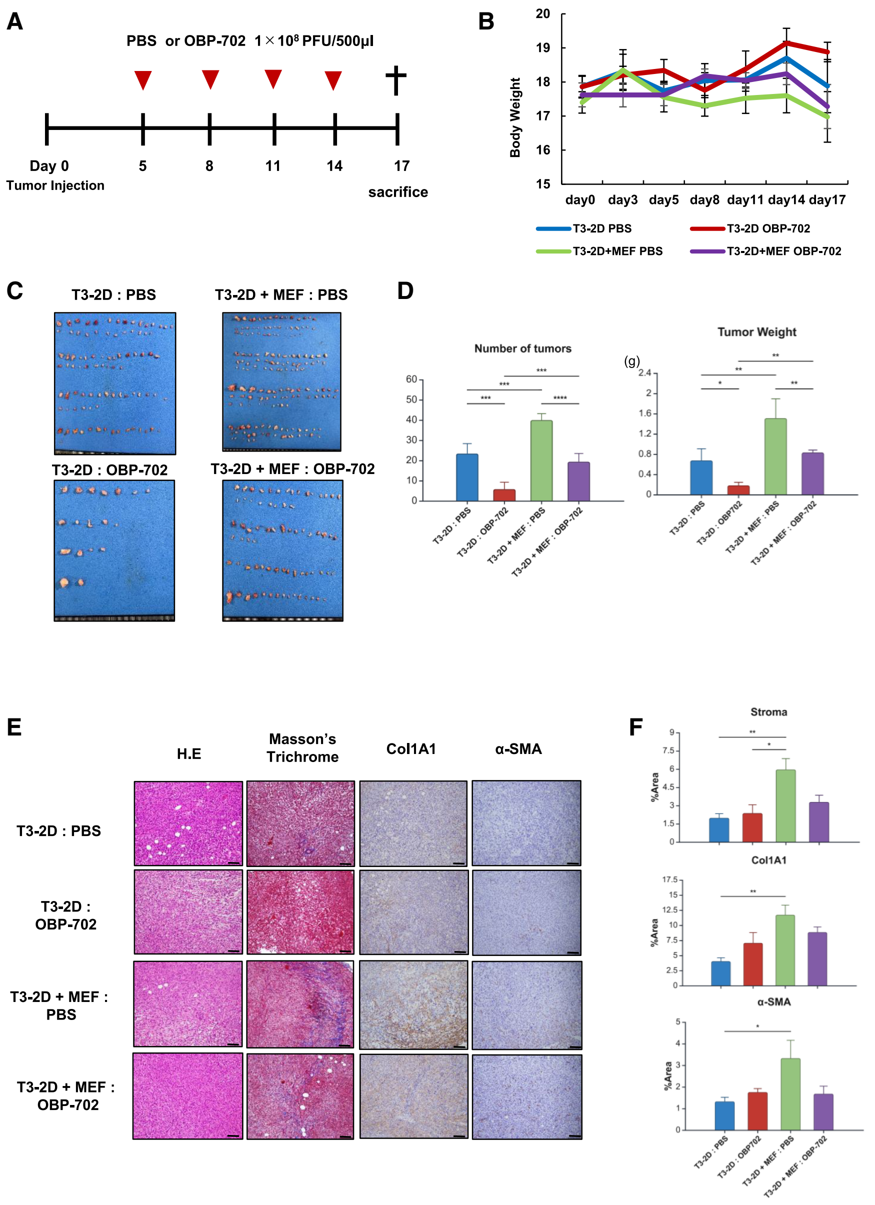

Figure 4. Antitumor effect of OBP-702 was attenuated in collagen-rich PM of GC

T3-2D (5×10^4 cells) cells alone and those co-inoculated with MEF (5×10^5 cells) were inoculated into the abdominal cavity of C57BL/6J mice and treated with intraperitoneal administration of PBS or OBP-702 (1×10^8 PFU/body) four times. (A) Schema of the treatment schedule. Red arrowheads show the timing of treatment with OBP-702 and the black cross indicates sacrifice. (B) Body weight changes in mice of each group. (C) Macroscopic images of peritoneal nodules in each group. (D) The total number and weight of PMs were measured 17 days after tumor inoculation. Data are shown as the mean value ±SD ($n = 4$). (E) Representative microscopic images with H&E, Masson trichrome, Col1A1, and α-SMA staining of peritoneal nodules in each group. Scale bars, 100 μm. (F) Area indexes of stroma, Col1A1, and α-SMA in each staining was evaluated by ImageJ software. The mean area index was calculated from three selected fields in each mouse, from a total of four mice per group. Data are expressed as the mean ± SD ($n = 4$). $^{*}p < 0.05$, $^{**}p < 0.01$, $^{***}p < 0.001$, $^{****}p < 0.0001$.

desmoplasia in pancreatic cancer in preclinical models.[44] Furthermore, we have recently demonstrated that CAFs enhanced the malignancy of pancreatic cancer cells by increasing the secretion of IL-8 from neutrophils, and proposed that PFD might suppress the malignant potential of cancer cells by inhibition of CAFs.[45] However, no report has investigated whether PFD could enhance the antitumor effects of OV.

We have previously demonstrated that OBP-702 infects CAFs in addition to cancer cells, and induces cell death by the induction of wild-type p53.[20] However, the present study showed that low doses of OBP-702 were insufficient to suppress production of collagen by CAFs. PFD could enhance the penetration of OBP-702 to the interior of spheroids by suppressing CAF activation and collagen production. Moreover, in the mouse model of PM, PFD suppressed collagen production within the PM tumors, and intraperitoneal treatment of OBP-702 combined with PFD significantly suppressed the formation of PM compared to OBP-702 monotherapy. In the combination treatment group, OBP-702 could penetrate deeply within PM tumors, which suggests that suppression of collagen production by PFD might have improved the penetration of OBP-702.

The novel therapeutic approach of targeting CAFs is a promising strategy in the treatment of PM of GC. IL-6 secreted by CAFs and TAMs in the TME is associated with tumor immunosuppression, we have demonstrated that inhibiting IL-6 suppressed the development of PM of GC.[46,47] Furthermore, we have previously found that alteration of p53 phosphorylation in CAFs contributed to its cancer-supportive properties; and reported that intraperitoneal administration of OBP-702 (which causes wild-type p53 overexpression) had therapeutic effects against CAFs in addition to cancer cells, and synergistically suppressed PM in combination with paclitaxel.[20] Moreover, intraperitoneal CD163+ TAM is associated with the development and progression of PM of GC, and OBP-702 could cause remodeling of intraperitoneal macrophages to the inflammatory phenotype, M1 macrophage.[23] PFD could enhance the antitumor effects of OBP-702 and restore antitumor immunity by depleting collagen secreted by CAFs. The safety and feasibility of intraperitoneal administration of OV in patients

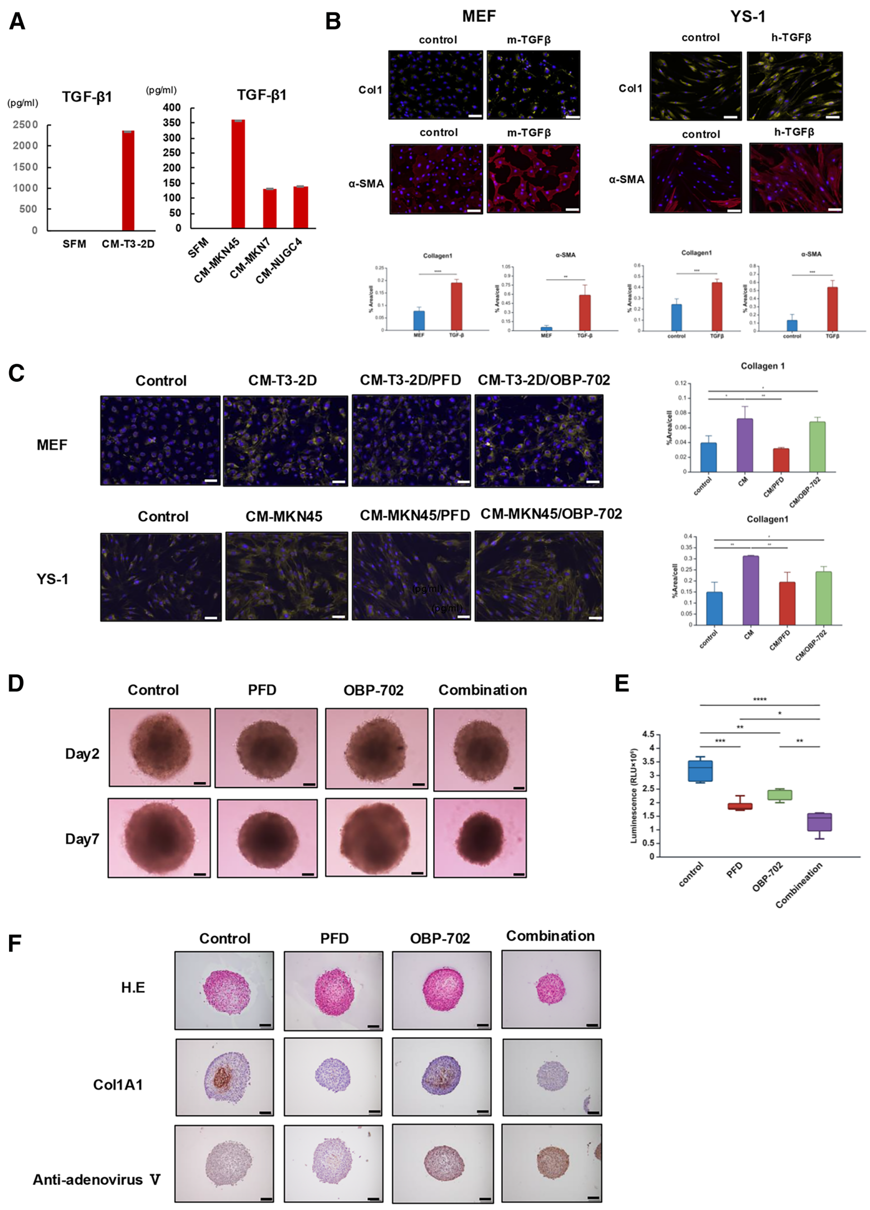

Figure 5. Pirfenidone (PFD) decreased collagen expression in CAFs and enhanced the antitumor effect of OBP-702 on spheroid models

(A) The concentration of TGF-β in the CM of each GC cell was analyzed using ELISA. SFM was used as a control. (B) Representative images of immunocytochemical staining of collagen 1 and α-SMA in MEF and YS-1 cells after incubation with recombinant TGF-β for 4 days. SFM was used as a control. The area index for each staining was evaluated by ImageJ software. Data are expressed as the mean ± SD (n = 3). Scale bars, 100 μm. (C) Representative images of immunocytochemical staining of collagen 1 in MEF and YS-1 cells after incubation with CM of T3-2D or MKN45 cells treated with PFD (1 mM) or OBP-702 (20 MOI or 50 MOI) for 4 days. SFM was used as a control. The area index for each staining was evaluated by ImageJ software. Data are expressed as the mean ± SD (n = 3). Scale bars, 100 μm. (D) Representative images of T3-2D spheroids co-cultured with MEF after incubation with PFD (1 mM), OBP-702 (20 MOI), or in combination, for 7 days. (E) ATP cell viability assay of T3-2D and MEF co-culture of spheroids after treated with PFD (1 mM), OBP-702 (20 MOI), or in combination, for 7 days. Data are expressed as the mean ± SD (n = 5). (F) Representative microscopic images with H&E, Col1A1 and anti-adenovirus type V staining of co-culture spheroids treated with PFD (1 mM), OBP-702 (20MOI) or in combination, for 7 days. Scale bars, 100 μm. *$p < 0.05$, **$p < 0.01$, ***$p < 0.001$, ****$p < 0.0001$.

with advanced ovarian cancer have been confirmed in a phase I clinical trial.[48] The safety and feasibility of intratumoral injection of OBP-301 also confirmed in phase I clinical trials.[49] PFD is clinically used as an oral medication for IPF, and its safety has been established. Therefore, the combination therapy of intraperitoneal treatment with OBP-702 and PFD may be a novel therapeutic strategy for PM of GC.

This study has some limitations. First, in the co-culture spheroid model of cancer cells and fibroblasts, fibroblasts aggregated in the center of the spheroids as time passes, which did not fully mimic *in vivo* and clinical situations. However, the collagen produced by CAFs is involved in the proliferation of cancer cells and inhibiting the penetration of OV into the tumors. Second, there was some variation in the size of the peritoneal nodules in the same treatment. Intraabdominal administration of OV could not spread evenly through the abdominal cavity. Therefore, it is necessary to consider the method of administration of OV and the position of the patients after administration.

In conclusion, we have demonstrated that higher collagen expression in advanced GC tissues is associated with poor prognosis and with development of PM. PFD could enhance the penetration of OBP-702 to the interior of tumors by collagen depletion in PM of GC. Therefore, combination therapy of OBP-702 and PFD has potential as a novel therapeutic strategy for PM of GC.

MATERIALS AND METHODS

Patients and IHC analysis of clinical samples

A total of 106 patients with GC categorized as having subserosal (SS) or serosal (SE) invasion who received gastrectomy at Okayama University Hospital between 2011 and 2015 were retrospectively reviewed. Table S1 lists the patients' characteristics. Twenty patients with PM of GC who underwent diagnostic resection of peritoneal nodules between 2014 and 2019 were also investigated. This study

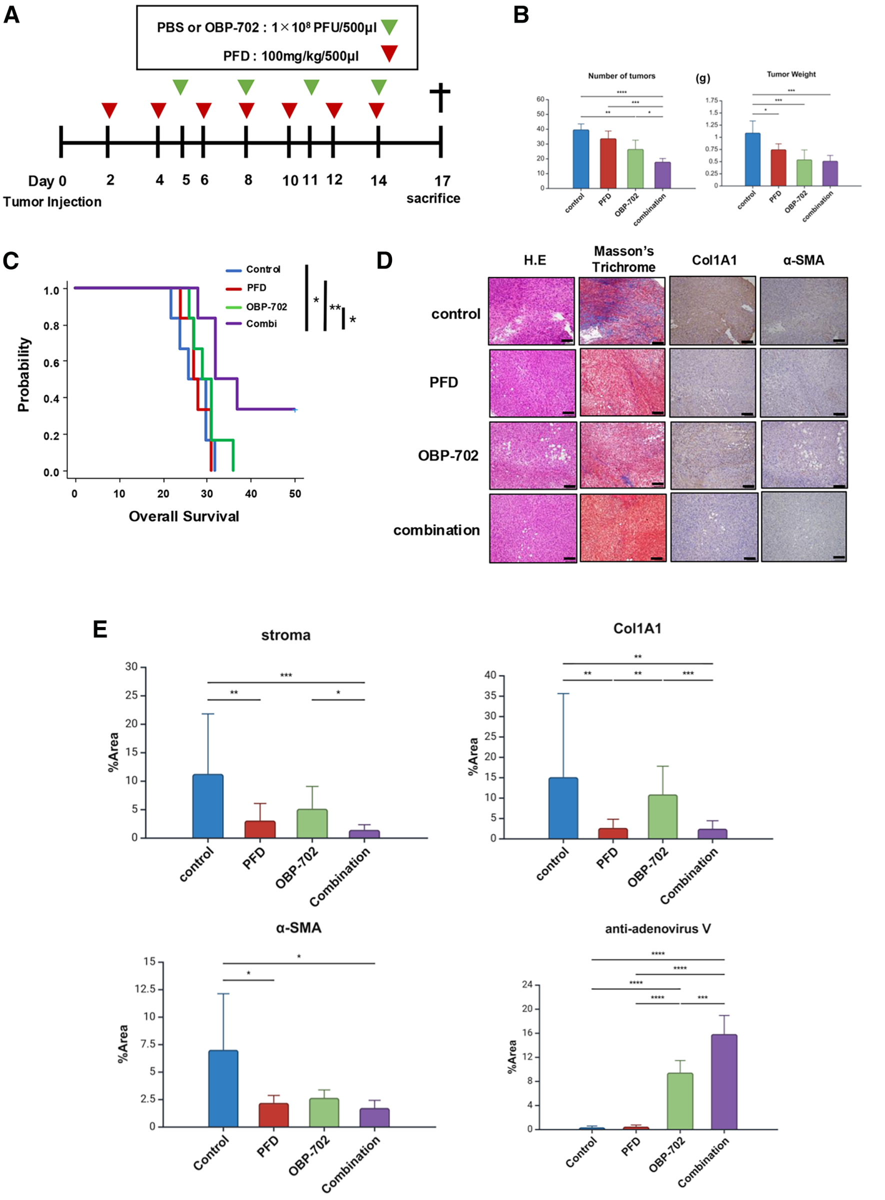

Figure 6. Collagen depletion by pirfenidone (PFD) enhances the antitumor effects of OBP-702 in peritoneal metastasis

(A) Schema of the treatment schedule. Orthotopic mouse peritoneal metastasis model of T3-2D and MEF co-injection treated by intraperitoneal administration of PBS, PFD, OBP-702, or the combination of PFD and OBP-702. Green arrowheads show the timing of treatments with PBS or OBP-702, and red arrowheads show treatments with PFD. The black cross indicates sacrifice. (B) The total number and weight of PM were measured 17 days after tumor inoculation. Data are shown as the mean value ±SD (n = 5). (C) Kaplan-Meier curves for overall survival in mice treated with each treatment. (D) Representative microscopic images with H&E, Masson trichrome, Col1A1, α-SMA, and anti-adenovirus type V staining of peritoneal nodules in each group. Scale bars, 100 μm. (E) Area indexes of stroma, Col1A1, α-SMA, and adenovirus type V in the tumors were evaluated by ImageJ software. The mean area index was calculated from three selected fields in each mouse, from a total of five mice per group. Data are expressed as the mean ± SD (n = 5). *$p < 0.05$, **$p < 0.01$, ***$p < 0.001$, ****$p < 0.0001$.

was approved by the Institutional Review Board of Okayama University (No. 2208-032). After confirming the presence of tumor using hematoxylin and eosin staining, Masson's trichrome staining was performed using trichrome stain kit (Modified Masson's) (ScyTek Laboratories, Logan, UT), according to the manufacturer's instructions. Sectioned tissues were incubated with mouse anti-alpha-smooth muscle actin (α-SMA) monoclonal antibody (mAb) (a5228, Sigma-Aldrich, St. Louis, MO) for immunohistochemistry. Immunoreactive signals were visualized with a 3,3′-diaminobenzidine tetra hydrochloride solution, and nuclei were counterstained with hematoxylin. The expression levels of collagen and α-SMA were evaluated using area index, calculated at low magnification (X 100) by ImageJ software (http://rsb.info.nih.gov/ij/), as described previously.[20] For each case, four different fields were randomly selected from the advancing edge of the cancer cells, and the expression levels of collagen and α-SMA were measured in the same fields. The area index for each of collagen and α-SMA was calculated as the mean value in each sectioned tissue. All evaluations were performed by an independent pathologist blinded to the clinical information. Sections were observed under light microscopy (BX50; Olympus, Tokyo, Japan).

Public datasets

The Kaplan-Meier plotter (Kaplan-Meier plotter [Gastric cancer]) was used to evaluate the relationship between the expression levels of collagen 1 related genes (Col1A1 and Col1A2) and overall survival (OS) in GC patients. Gene expression profiles for Col1A1 and Col1A2 were retrieved from public datasets, including the GSE15459, GSE22377, and GSE62254 datasets. The "auto-select best cutoff" option was utilized to automatically determine the optimal cutoff point for dichotomizing expression levels into high and low groups. This selection was based on maximizing the statistical significance of survival differences between groups. The log rank test was employed to assess statistical differences between survival distributions. Statistical significance was set at $p < 0.05$. Hazard ratios and corresponding 95% confidence intervals were calculated using Cox proportional hazards regression to validate the survival differences observed in the Kaplan-Meier analysis.

Cell lines

Three human gastric cancer cell lines (MKN7, MKN45, and NUGC-4) were purchased from the Japanese Collection of Research Bioresources (JCRB) Cell Bank and maintained in RPMI-1640 medium or DMEM supplemented with 10% heat-inactivated fetal bovine serum (FBS) (Sigma-Aldrich, St. Louis, MO). NUGC-4 cells expressing red fluorescent protein (RFP) were obtained from AntiCancer, Inc. (San Diego, CA). T3-2D, a murine gastric cancer cell line established by Dr. Ohki at the National Cancer Center Research Institute,[50] was kindly provided and maintained in DMEM supplemented with 10% FBS. This study used two human fibroblasts and one murine fibroblast. YS-1, a human primary cell line derived from tumor-infiltrating fibroblasts of the stomach, was purchased from the JCRB Cell Bank and maintained in a 1:1 mixture of Ham's F-12 with L-glutamine and phenol red and DMED supplemented with 5% FBS. FEF3, a primary human esophageal fibroblast, was isolated from human fetal esophagus as described previously[51] and maintained in DMEM supplemented with 10% FBS. A murine embryonic fibroblast (MEF) cell line was purchased from the American Type Culture Collection (Manassas, VA) and maintained in DMEM supplemented with 15% FBS. All media were supplemented with 100 U/mL penicillin and 100 μg/mL streptomycin. Cells were routinely maintained at 37°C in a humidified atmosphere with 5% CO_2.

Recombinant adenovirus and reagents

The recombinant, telomerase-specific, replication-competent adenovirus (Ad) vector, OBP-301 (suratadenoturev), has been described and characterized elsewhere.[13,15] OBP-401 (TelomeScan) is a telomerase-specific, replication-competent Ad variant into which the replication cassette and green fluorescence protein (GFP) expression under control of the cytomegalovirus promoter were inserted into the E3 region in OBP-301 for monitoring of viral replication.[52] OBP-702 is another adenovirus variant that inserts a human wild-type p53 gene expression cassette under the control of the Egr-1 promoter into the E3 region of OBP-301.[18,19] Viruses were purified by ultracentrifugation using CsCl step gradients. Viral titers were determined by plaque-forming assay using 293 cells, and the virus was stored at −80°C. 3D Ready Atelocollagen DMEM low glucose was obtained from KOKEN CO., LTD. (Tokyo, Japan). Recombinant human transforming growth factor β1 (TGF-β1) was obtained from Sigma-Aldrich. Recombinant murine TGF-β1 was obtained from R&D systems Inc. (Minneapolis, MN). PFD was obtained from MedChemExpress (Monmouth Junction, NJ) and ultrasonically diluted in PBS for the *in vivo* and *in vitro* experiments.

Spheroid culture

Spheroids were constructed according to the liquid overlay technique using 96-well ultra-low attachment plates (PrimeSurface plate96M, Sumitomo Bakelite Co., Ltd. Tokyo, Japan). For construction of mono-type spheroids, GC cells were seeded at a density of 5×10^3 cells/100 μL/well into each well and cultured for 4 to 7 days. For the construction of co-culture-type spheroids of GC cells and fibroblasts, GC cells were seeded at a density of 5×10^3 cells/100 μL/well and fibroblasts were seeded at a density of 1×10^4 cells/100 μL/well into each well and cultured for 4 to 7 days.[53] Collagen 1 was added to the culture medium to constitute 2%. GC cells were stained with 10 mM CellTracker Red CMTPX Dye (Invitrogen, Carlsbad, CA) and fibroblasts were stained with 10 mM CellTracker Green CMFDA Dye (Invitrogen) or CellTracker Blue CMAC Dye (Invitrogen) for 30 min at 37°C in the absence of FBS. Spheroids were photographed using a confocal microscope (APX100; Olympus, Tokyo, Japan), and analyzed using imaging software (cellSens; Olympus).

Conditioned medium preparation

To collect conditioned medium (CM) from human and murine GC cells, each GC cell was seeded in a 100 mm dish at a density of 1×10^6 cells/dish and incubated for 24 h. After washing with phosphate-buffered saline (PBS), serum-free medium (SFM) was added and cells were incubated for 48 h. After centrifugation, the supernatant was collected as CM.

Activation of CAFs

Human and murine fibroblasts were seeded at a density of 1×10^4 cells/mL and incubated for 24 h. The culture medium was changed to a CM and incubated for 96 h. To evaluate the role of TGF-β1 in the activation of CAFs, all fibroblasts were seeded the same way. After 24 h, the cells were incubated for 5 days with human or murine recombinant TGF-β1.

Cell viability assay

Human (MKN45 and YS-1) and murine (T3-2D and MEF) cells were seeded on 96-well plates at a density of 1 or 5×10^3 cells/well and cultured for 24 h before viral infection or administration of PFD. All cells were infected with OBP-702 at multiplicity of Infections (MOIs) of 0, 10, 20, 50, 100, or 200 plaque-forming units (PFU)/cell for 72 h, or treated with PFD at concentrations of 0, 0.5, 1, 2.5, 5, or 10 mM for 72 h. Collagen 1 was added to the culture medium to constitute 10%. Cell viability was examined using the cell proliferation kit II (Roche Diagnostics GmbH, Mannheim, Germany), which is based on the sodium 3'-[1-(phenylaminocarbonyl)-3,4-tetrazolium]-bis(4-methoxy-6-nitro) benzene sulfonic acid hydrate (XTT) assay in accordance with the manufacturer's protocol.

Cell proliferation assay in collagen containing medium

T3-2D and MKN45 cells were seeded on 96-well plates at a density of 1 or 5×10^3 cells/well and cultured for 24 h. The culture medium was changed to collagen 1-containing medium at a concentration of 0% or 10%. Cell viability was determined using the XTT assay, examined at OD 450 nm on a microplate reader.

Quantitative real-time PCR analysis

Total RNA was isolated from cells using the RNeasy Mini Kits (QIAGEN, Hilden, Germany), according to the manufacturer's instructions. The cDNA was synthesized from 1.0 mg of total RNA using Advantage RT-for-PCR Kit (Clontech Laboratories, Mountain View, CA). Quantitative real-time PCR was performed for gene expression analysis using the StepOnePlus Real-Time PCR System

(Applied Biosystems, Waltham, MA) with TaqMan PCR Master Mix (Applied Biosystems, Foster City, CA) for murine cell lines or SYBR Green Master Mix (Life Technologies, Carlsbad, CA) for human cell lines. The primers were murine GAPDH (Mm99999915_g1, Applied Biosystems), murine Col1A1 (Mm00801666_g1, Applied Biosystems), murine Col1A2 (Mm00483888_m1, Applied Biosystems), murine ACTA2 (Mm00725412_s1, Applied Biosystems), human GAPDH (Integrated Device Technology, Coralville, IA), human Col1A1 (Integrated Device Technology), human Col1A2 (Integrated Device Technology), and human ACTA2 (Integrated Device Technology). GAPDH was used as a normalization control. The relative expression of each mRNA was determined using the $2^{-\Delta\Delta Ct}$ method. Primer sequences are shown in Table S2.

Immunofluorescence

Fibroblasts were seeded at a density of 1 $\times 10^4$ cells/mL for 24 h. Spheroids were harvested from a 96-well plate and processed in a 1.5 mL Eppendorf tube. Following three washes with PBS, cells were fixed in 100% methanol for 30 min at room temperature. After blocking endogenous peroxidases, cells were incubated with primary antibody; rabbit anti-collagen 1 polyclonal antibody (pAb) (ab34710, 1:200; Abcam, Melbourne, VIC, Australia), rabbit anti-collagen 1 pAb (ab21286, 1:200; Abcam), rabbit anti-α-SMA monoclonal antibody (mAb) (19245, 1:500; Cell Signaling Technology, Danvers, MA), rabbit anti-adenovirus type 5 pAb (ab6982, 1:800; Abcam) in 3% BSA overnight at 4°C. Following three washes with PBS, cells were incubated with Alexa Fluor 647–conjugated goat anti-mouse IgG pAb (Invitrogen) as the secondary antibody for 60 min at 4°C. After washing, nuclei were stained with DAPI (Invitrogen) for 3 min. Cells were photographed using a fluorescence microscope (IX83; Olympus) and analyzed using imaging software (cellSens; Olympus).

ELISA

Cells were seeded at a density of 4 $\times 10^4$ cells/mL and cultured for 24 h. After washing with PBS, SFM was added, and supernatants were collected after 48 h. The concentrations of TGF-β1 in CM were determined using Quantikine ELISA kits for human TGF-β1 (#DB100B; R&D Systems, Minneapolis, MN) according to the manufacturer's protocol.

Animal experiments

T3-2D cells (5 $\times 10^4$ cells) were inoculated into the peritoneal cavity of 6- to 8-week-old female C57BL/6 mice (CLEA Japan, Tokyo, Japan) as cancer cell single-injection models of PM of GC. In the co-injection model, both T3-2D (5 $\times 10^4$ cells) and MEF (5 $\times 10^5$ cells) were inoculated into the peritoneal cavity. Five days after cell inoculation, 500 μL of solution containing OBP-702 (1 $\times$ 10^8 PFU) or PBS was injected into the intraperitoneal cavity every 3 days for a total of 4 doses in the OBP-702 monotherapy experiment. All tumor nodules in the peritoneal cavity were resected and total weights were measured on day 17. In the combination therapy, 2 days before OBP-702 injection, 100 mg/body weight of PFD was injected intraperitoneally every 2 days for a total of 7 times, and 4 doses of OBP-702 in total were injected intraperitoneally. Intraperitoneal administration of PFD was performed as described previously.[54] Five or six mice were used in each group. All tumor nodules in the peritoneal cavity were resected and the total weights were measured on day 17. Survival duration was monitored, and overall survival was calculated using a model in which both T3-2D (5 $\times 10^3$ cells) and MEF (5 $\times 10^4$ cells) were inoculated into the peritoneal cavity and the same treatment protocol was applied.

Immunohistochemistry

For histological analyses, mouse peritoneal tumor nodules were removed and fixed in 10% neutralized formalin. All tissues were subsequently dehydrated in alcohol and embedded in paraffin block. Tissue sections (4 μm) were deparaffinized in xylene and rehydrated in a graded ethanol series. After blocking endogenous peroxidases by incubation with 3% H_2O_2 for 10 min, the samples were boiled in citrate buffer or EDTA buffer for 14 min in a microwave oven for antigen retrieval. Samples were incubated with primary antibodies overnight at 4°C and then with peroxidase-linked secondary antibody for 30 min at room temperature. Primary antibodies against rabbit anti-Col1A1 mAb (E8F4L, 1:200; Cell Signaling Technology), rabbit anti-α-SMA mAb (19245, 1:500; Cell Signaling Technology), mouse anti-CD8a mAb (4SM15, 1:200; eBioscience, San Diego, CA), rabbit anti-CD163 mAb (ab182422, 1:500; Abcam), and rabbit anti-adenovirus type VpAb (ab6982, 1:800; Abcam) were used. Immunoreactive signals were visualized with a 3,39-diaminobenzidine tetrahydrochloride solution and the nuclei were counterstained with hematoxylin. Masson's trichrome staining was performed using the trichrome stain kit (Modified Masson's) (ScyTek Laboratories), according to the manufacturer's instructions. Sections were viewed under a microscope (BX50; Olympus).

Statistical analysis

For the area indexes of collagen and α-SMA, cutoff was defined using the median value of the high or low groups. OS and relapse-free survival (RFS) was calculated using the Kaplan-Meier method, with the log rank test used for comparisons between subgroups. Correlations between the two groups were examined using Spearman's rank correlation coefficient. Student's t test was used to compare differences in means between two groups, and analysis of variance with Tukey's test was used to compare differences in means between multiple groups. All data are expressed as the mean ± SD. Values of $p < 0.05$ were considered statistically significant. Statistical analysis and graph creation were performed using GraphPad Prism software.

Study approval

This study was conducted in accordance with the ethical standards of the Declaration of Helsinki and the ethical guidelines for medical and health research involving human subjects. Studies using clinical samples were approved and reviewed by the institutional review board of Okayama University Hospital (approval No. 2208-032). All animal experimental protocols were approved by the Ethics Review Committee for Animal Experiments of Okayama University. All animal experimental protocols were approved by the Ethics Review

Committee for animal experiments of Okayama University (approval no. OKU-2023143, 2023146).

DATA AVAILABILITY

All data generated or analyzed during this study are included in this article and its supplemental information files. Further enquiries can be directed to the corresponding author.

ACKNOWLEDGMENTS

This work was supported by JSPS KAKENHI (grant no. 24K11912) (S.Ki.). We wish to thank Ms. Tomoko Sueishi, Ms. Tae Yamanishi, and Ms. Yuko Hoshijima for their excellent technical assistance.

AUTHOR CONTRIBUTIONS

Conception and design: S.K., H.T., and T.F.

Development of methodology: T.O., S.K., H.T., and T.F.

Acquisition of data: T.O., E.M., Y.U., S.K., Y.M., and N.K.

Analysis and interpretation of data: T.O., S.K., H.T., T.O., K.N., and T.F.

Writing, review, and/or revision of the manuscript: T.O. and S.K.

Administrative, technical, or material support: J.O., R.O., and Y.U.

Study supervision: H.T., K.S., T.O., S.K., K.N., S.K., R.O., and T.F.

DECLARATION OF INTERESTS

Y.U. is the president and CEO of Oncolys BioPharma, Inc., the manufacturer and patent holder of OBP-702. H.T. and T.F. are consultants for Oncolys BioPharma, Inc.

SUPPLEMENTAL INFORMATION

Supplemental information can be found online at https://doi.org/10.1016/j.omton.2025.201045.

REFERENCES

1. Sung, H., Ferlay, J., Siegel, R.L., Laversanne, M., Soerjomataram, I., Jemal, A., and Bray, F. (2021). Global Cancer Statistics 2020: GLOBOCAN Estimates of Incidence and Mortality Worldwide for 36 Cancers in 185 Countries. CA Cancer J. Clin. *71*, 209–249.

2. Verstegen, M.H., Harker, M., van de Water, C., van Dieren, J., Hugen, N., Nagtegaal, I.D., Rosman, C., and van der Post, R.S. (2020). Metastatic pattern in esophageal and gastric cancer: Influenced by site and histology. World J. Gastroenterol. *26*, 6037–6046.

3. Monster, J.L., Kemp, L.J.S., Gloerich, M., and van der Post, R.S. (2022). Diffuse gastric cancer: Emerging mechanisms of tumor initiation and progression. Biochim. Biophys. Acta. Rev. Cancer *1877*, 188719.

4. Ge, S., Xia, X., Ding, C., Zhen, B., Zhou, Q., Feng, J., Yuan, J., Chen, R., Li, Y., Ge, Z., et al. (2018). A proteomic landscape of diffuse-type gastric cancer. Nat. Commun. *9*, 1012.

5. Jinawath, N., Furukawa, Y., Hasegawa, S., Li, M., Tsunoda, T., Satoh, S., Yamaguchi, T., Imamura, H., Inoue, M., Shiozaki, H., and Nakamura, Y. (2004). Comparison of gene-expression profiles between diffuse- and intestinal-type gastric cancers using a genome-wide cDNA microarray. Oncogene *23*, 6830–6844.

6. Liu, X., Yao, L., Qu, J., Liu, L., Lu, N., Wang, J., and Zhang, J. (2021). Cancer-associated fibroblast infiltration in gastric cancer: the discrepancy in subtypes pathways and immunosuppression. J. Transl. Med. *19*, 325.

7. Ishimoto, T., Miyake, K., Nandi, T., Yashiro, M., Onishi, N., Huang, K.K., Lin, S.J., Kalpana, R., Tay, S.T., Suzuki, Y., et al. (2017). Activation of Transforming Growth Factor Beta 1 Signaling in Gastric Cancer-associated Fibroblasts Increases Their Motility, via Expression of Rhomboid 5 Homolog 2, and Ability to Induce Invasiveness of Gastric Cancer Cells. Gastroenterology *153*, 191–204.e16.

8. Nissen, N.I., Karsdal, M., and Willumsen, N. (2019). Collagens and Cancer associated fibroblasts in the reactive stroma and its relation to Cancer biology. J. Exp. Clin. Cancer Res. *38*, 115.

9. Li, J., Ding, Y., and Li, A. (2016). Identification of COL1A1 and COL1A2 as candidate prognostic factors in gastric cancer. World J. Surg. Oncol. *14*, 297.

10. Chen, D., Liu, Z., Liu, W., Fu, M., Jiang, W., Xu, S., Wang, G., Chen, F., Lu, J., Chen, H., et al. (2021). Predicting postoperative peritoneal metastasis in gastric cancer with serosal invasion using a collagen nomogram. Nat. Commun. *12*, 179.

11. Wang, J., Liu, Z., Lin, L., Wu, Z., Gao, X., Cai, X., Chang, L., Xia, X., Zhang, H., and Chen, G. (2023). Collagen-related gene expression level predicts the prognosis and immune therapy response. Gastric Cancer *26*, 891–903.

12. Shao, S., Yang, X., Zhang, Y.N., Wang, X.J., Li, K., Zhao, Y.L., Mou, X.Z., and Hu, P. Y. (2022). Oncolytic Virotherapy in Peritoneal Metastasis Gastric Cancer: The Challenges and Achievements. Front. Mol. Biosci. *9*, 835300.

13. Kawashima, T., Kagawa, S., Kobayashi, N., Shirakiya, Y., Umeoka, T., Teraishi, F., Taki, M., Kyo, S., Tanaka, N., and Fujiwara, T. (2004). Telomerase-specific replication-selective virotherapy for human cancer. Clin. Cancer Res. *10*, 285–292.

14. Hashimoto, Y., Watanabe, Y., Shirakiya, Y., Uno, F., Kagawa, S., Kawamura, H., Nagai, K., Tanaka, N., Kumon, H., Urata, Y., and Fujiwara, T. (2008). Establishment of biological and pharmacokinetic assays of telomerase-specific replication-selective adenovirus. Cancer Sci. *99*, 385–390.

15. Sasaki, T., Tazawa, H., Hasei, J., Kunisada, T., Yoshida, A., Hashimoto, Y., Yano, S., Yoshida, R., Uno, F., Kagawa, S., et al. (2011). Preclinical evaluation of telomerase-specific oncolytic virotherapy for human bone and soft tissue sarcomas. Clin. Cancer Res. *17*, 1828–1838.

16. Ishikawa, W., Kikuchi, S., Ogawa, T., Tabuchi, M., Tazawa, H., Kuroda, S., Noma, K., Nishizaki, M., Kagawa, S., Urata, Y., and Fujiwara, T. (2020). Boosting Replication and Penetration of Oncolytic Adenovirus by Paclitaxel Eradicate Peritoneal Metastasis of Gastric Cancer. Mol. Ther. Oncolytics *18*, 262–271.

17. Nemunaitis, J., Tong, A.W., Nemunaitis, M., Senzer, N., Phadke, A.P., Bedell, C., Adams, N., Zhang, Y.A., Maples, P.B., Chen, S., et al. (2010). A phase I study of telomerase-specific replication competent oncolytic adenovirus (telomelysin) for various solid tumors. Mol. Ther. *18*, 429–434.

18. Yamasaki, Y., Tazawa, H., Hashimoto, Y., Kojima, T., Kuroda, S., Yano, S., Yoshida, R., Uno, F., Mizuguchi, H., Ohtsuru, A., et al. (2012). A novel apoptotic mechanism of genetically engineered adenovirus-mediated tumour-specific p53 overexpression through E1A-dependent p21 and MDM2 suppression. Eur. J. Cancer *48*, 2282–2291.

19. Hasei, J., Sasaki, T., Tazawa, H., Osaki, S., Yamakawa, Y., Kunisada, T., Yoshida, A., Hashimoto, Y., Onishi, T., Uno, F., et al. (2013). Dual programmed cell death pathways induced by p53 transactivation overcome resistance to oncolytic adenovirus in human osteosarcoma cells. Mol. Cancer Ther. *12*, 314–325.

20. Ogawa, T., Kikuchi, S., Tabuchi, M., Mitsui, E., Une, Y., Tazawa, H., Kuroda, S., Noma, K., Ohara, T., Kagawa, S., et al. (2022). Modulation of *p53* expression in cancer-associated fibroblasts prevents peritoneal metastasis of gastric cancer. Mol. Ther. Oncolytics *25*, 249–261.

21. Hori, N., Tazawa, H., Li, Y., Okura, T., Kikuchi, S., Kuroda, S., Ohara, T., Noma, K., Nishizaki, M., Urata, Y., et al. (2023). Intraperitoneal Administration of p53-armed Oncolytic Adenovirus Inhibits Peritoneal Metastasis of Diffuse-type Gastric Cancer Cells. Anticancer Res. *43*, 4809–4821. https://doi.org/10.21873/anticanres.

22. Kaufman, H.L., Kohlhapp, F.J., and Zloza, A. (2015). Oncolytic viruses: a new class of immunotherapy drugs. Nat. Rev. Drug Discov. *14*, 642–662.

23. Tabuchi, M., Kikuchi, S., Tazawa, H., Okura, T., Ogawa, T., Mitsui, E., Une, Y., Kuroda, S., Sato, H., Noma, K., et al. (2024). Functional remodeling of intraperitoneal macrophages by oncolytic adenovirus restores anti-tumor immunity for peritoneal metastasis of gastric cancer. Mol. Ther. Oncol. *32*, 200806.

24. Yoon, A.R., Hong, J., Jung, B.K., Ahn, H.M., Zhang, S., and Yun, C.O. (2023). Oncolytic adenovirus as pancreatic cancer-targeted therapy: Where do we go from here? Cancer Lett. *579*, 216456.

25. Ruwanpura, S.M., Thomas, B.J., and Bardin, P.G. (2020). Pirfenidone: Molecular Mechanisms and Potential Clinical Applications in Lung Disease. Am. J. Respir. Cell Mol. Biol. *62*, 413–422.
26. Peng, D., Fu, M., Wang, M., Wei, Y., and Wei, X. (2022). Targeting TGF-β signal transduction for fibrosis and cancer therapy. Mol. Cancer *21*, 104.
27. Cai, T., Jiang, J., Yao, W., Hu, Y., Kong, S., Fan, Q., Yan, X., Li, F., and Shi, Z. (2023). Pirfenidone inhibits stromal collagen deposition and improves intra-tumoral delivery and antitumor efficacy of Pegylated liposomal doxorubicin. Biomed. Pharmacother. *157*, 114015.
28. Lei, Y., Xu, J., Xiao, M., Wu, D., Xu, H., Yang, J., Mao, X., Pan, H., Yu, X., and Shi, S. (2024). Pirfenidone alleviates fibrosis by acting on tumour-stroma interplay in pancreatic cancer. Br. J. Cancer *130*, 1505–1516.
29. Zhou, P., Du, X., Jia, W., Feng, K., and Zhang, Y. (2024). Engineered extracellular vesicles for targeted reprogramming of cancer-associated fibroblasts to potentiate therapy of pancreatic cancer. Signal Transduct. Target. Ther. *9*, 151.
30. Quail, D.F., and Joyce, J.A. (2013). Microenvironmental regulation of tumor progression and metastasis. Nat. Med. *19*, 1423–1437.
31. Sahai, E., Astsaturov, I., Cukierman, E., DeNardo, D.G., Egeblad, M., Evans, R.M., Fearon, D., Greten, F.R., Hingorani, S.R., Hunter, T., et al. (2020). A framework for advancing our understanding of cancer-associated fibroblasts. Nat. Rev. Cancer *20*, 174–186.
32. Song, K., Yu, Z., Zu, X., Li, G., Hu, Z., and Xue, Y. (2022). Collagen Remodeling along Cancer Progression Providing a Novel Opportunity for Cancer Diagnosis and Treatment. Int. J. Mol. Sci. *23*, 10509.
33. Chen, Y., Kim, J., Yang, S., Wang, H., Wu, C.J., Sugimoto, H., LeBleu, V.S., and Kalluri, R. (2021). Type I collagen deletion in αSMA^{+} myofibroblasts augments immune suppression and accelerates progression of pancreatic cancer. Cancer Cell *39*, 548–565.e6.
34. Yashiro, M., and Hirakawa, K. (2010). Cancer-stromal interactions in scirrhous gastric carcinoma. Cancer Microenviron. *3*, 127–135.
35. Li, M., Wang, J., Wang, C., Xia, L., Xu, J., Xie, X., and Lu, W. (2020). Microenvironment remodeled by tumor and stromal cells elevates fibroblast-derived COL1A1 and facilitates ovarian cancer metastasis. Exp. Cell Res. *394*, 112153.
36. Chen, Y., Yang, S., Tavormina, J., Tampe, D., Zeisberg, M., Wang, H., Mahadevan, K. K., Wu, C.J., Sugimoto, H., Chang, C.C., et al. (2022). Oncogenic collagen I homotrimers from cancer cells bind to α3β1 integrin and impact tumor microbiome and immunity to promote pancreatic cancer. Cancer Cell *40*, 818–834.e9.
37. Zhang, J., Fu, L., Wang, H., Yonemura, A., Semba, T., Yasuda-Yoshihara, N., Nishimura, A., Tajiri, T., Tong, Y., Yasuda, T., et al. (2024). RAC1-mediated integrin alpha-6 expression in E-cadherin-deficient gastric cancer cells promotes interactions with the stroma and peritoneal dissemination. Cancer Lett. *591*, 216901.
38. McKee, T.D., Grandi, P., Mok, W., Alexandrakis, G., Insin, N., Zimmer, J.P., Bawendi, M.G., Boucher, Y., Breakefield, X.O., and Jain, R.K. (2006). Degradation of fibrillar collagen in a human melanoma xenograft improves the efficacy of an oncolytic herpes simplex virus vector. Cancer Res. *66*, 2509–2513.
39. Chen, Y., Hu, S., Shu, Y., Qi, Z., Zhang, B., Kuang, Y., Ma, J., and Cheng, P. (2022). Antifibrotic Therapy Augments the Antitumor Effects of Vesicular Stomatitis Virus Via Reprogramming Tumor Microenvironment. Hum. Gene Ther. *33*, 237–249.
40. Bazan-Peregrino, M., Garcia-Carbonero, R., Laquente, B., Álvarez, R., Mato-Berciano, A., Gimenez-Alejandre, M., Morgado, S., Rodríguez-García, A., Maliandi, M.V., Riesco, M.C., et al. (2021). VCN-01 disrupts pancreatic cancer stroma and exerts antitumor effects. J. Immunother. Cancer *9*, e003254.
41. Kiyokawa, J., Kawamura, Y., Ghouse, S.M., Acar, S., Barçın, E., Martínez-Quintanilla, J., Martuza, R.L., Alemany, R., Rabkin, S.D., Shah, K., and Wakimoto, H. (2021). Modification of Extracellular Matrix Enhances Oncolytic Adenovirus Immunotherapy in Glioblastoma. Clin. Cancer Res. *27*, 889–902.
42. Choi, I.K., Lee, Y.S., Yoo, J.Y., Yoon, A.R., Kim, H., Kim, D.S., Seidler, D.G., Kim, J. H., and Yun, C.O. (2010). Effect of decorin on overcoming the extracellular matrix barrier for oncolytic virotherapy. Gene Ther. *17*, 190–201.
43. Kim, J.H., Lee, Y.S., Kim, H., Huang, J.H., Yoon, A.R., and Yun, C.O. (2006). Relaxin expression from tumor-targeting adenoviruses and its intratumoral spread, apoptosis induction, and efficacy. J. Natl. Cancer Inst. *98*, 1482–1493.
44. Kozono, S., Ohuchida, K., Eguchi, D., Ikenaga, N., Fujiwara, K., Cui, L., Mizumoto, K., and Tanaka, M. (2013). Pirfenidone inhibits pancreatic cancer desmoplasia by regulating stellate cells. Cancer Res. *73*, 2345–2356.
45. Yagi, T., Kagawa, S., Nogi, S., Taniguchi, A., Yoshimoto, M., Suemori, K., Nagai, Y., Fujita, S., Kuroda, S., Kikuchi, S., et al. (2025). Cancer-associated fibroblasts promote pro-tumor functions of neutrophils in pancreatic cancer via IL-8: potential suppression by pirfenidone. Cancer Immunol. Immunother. *74*, 96.
46. Li, Y., Tazawa, H., Nagai, Y., Fujita, S., Okura, T., Shoji, R., Yamada, M., Kikuchi, S., Kuroda, S., Ohara, T., et al. (2024). Senescent Fibroblasts Potentiate Peritoneal Metastasis of Diffuse-type Gastric Cancer Cells *via* IL-8-mediated Crosstalk. Anticancer Res. *44*, 2497–2509.
47. Mitsui, E., Kikuchi, S., Okura, T., Tazawa, H., Une, Y., Nishiwaki, N., Kuroda, S., Noma, K., Kagawa, S., Ohara, T., et al. (2025). Novel treatment strategy targeting interleukin-6 induced by cancer associated fibroblasts for peritoneal metastasis of gastric cancer. Sci. Rep. *15*, 3267.
48. Galanis, E., Hartmann, L.C., Cliby, W.A., Long, H.J., Peethambaram, P.P., Barrette, B.A., Kaur, J.S., Haluska, P.J., Jr., Aderca, I., Zollman, P.J., et al. (2010). Phase I trial of intraperitoneal administration of an oncolytic measles virus strain engineered to express carcinoembryonic antigen for recurrent ovarian cancer. Cancer Res. *70*, 875–882.
49. Shirakawa, Y., Tazawa, H., Tanabe, S., Kanaya, N., Noma, K., Koujima, T., Kashima, H., Kato, T., Kuroda, S., Kikuchi, S., et al. (2021). Phase I dose-escalation study of endoscopic intratumoral injection of OBP-301 (Telomelysin) with radiotherapy in oesophageal cancer patients unfit for standard treatments. Eur. J. Cancer *153*, 98–108.
50. Ohtsuka, J., Oshima, H., Ezawa, I., Abe, R., Oshima, M., and Ohki, R. (2018). Functional loss of p53 cooperates with the in vivo microenvironment to promote malignant progression of gastric cancers. Sci. Rep. *8*, 2291.
51. Noma, K., Smalley, K.S.M., Lioni, M., Naomoto, Y., Tanaka, N., El-Deiry, W., King, A.J., Nakagawa, H., and Herlyn, M. (2008). The essential role of fibroblasts in esophageal squamous cell carcinoma-induced angiogenesis. Gastroenterology *134*, 1981–1993.
52. Kishimoto, H., Kojima, T., Watanabe, Y., Kagawa, S., Fujiwara, T., Uno, F., Teraishi, F., Kyo, S., Mizuguchi, H., Hashimoto, Y., et al. (2006). In vivo imaging of lymph node metastasis with telomerase-specific replication-selective adenovirus. Nat. Med. *12*, 1213–1219.
53. Lazzari, G., Nicolas, V., Matsusaki, M., Akashi, M., Couvreur, P., and Mura, S. (2018). Multicellular spheroid based on a triple co-culture: A novel 3D model to mimic pancreatic tumor complexity. Acta Biomater. *78*, 296–307.
54. Salah, M.M., Ashour, A.A., Abdelghany, T.M., Abdel-Aziz, A.A.H., and Salama, S.A. (2019). Pirfenidone alleviates concanavalin A-induced liver fibrosis in mice. Life Sci. *239*, 116982.

OMTON, Volume *33*

Supplemental information

Collagen depletion by pirfenidone enhances antitumor effect of oncolytic adenovirus against peritoneal metastases of gastric cancer

Tomohiro Okura, Satoru Kikuchi, Hiroshi Tazawa, Yu Mikane, Nobuhiko Kanaya, Ema Mitsui, Yuta Une, Kunitoshi Shigeyasu, Toshiaki Ohara, Shinji Kuroda, Kazuhiro Noma, Junko Ohtsuka, Rieko Ohki, Shunsuke Kagawa, Yasuo Urata, and Toshiyoshi Fujiwara

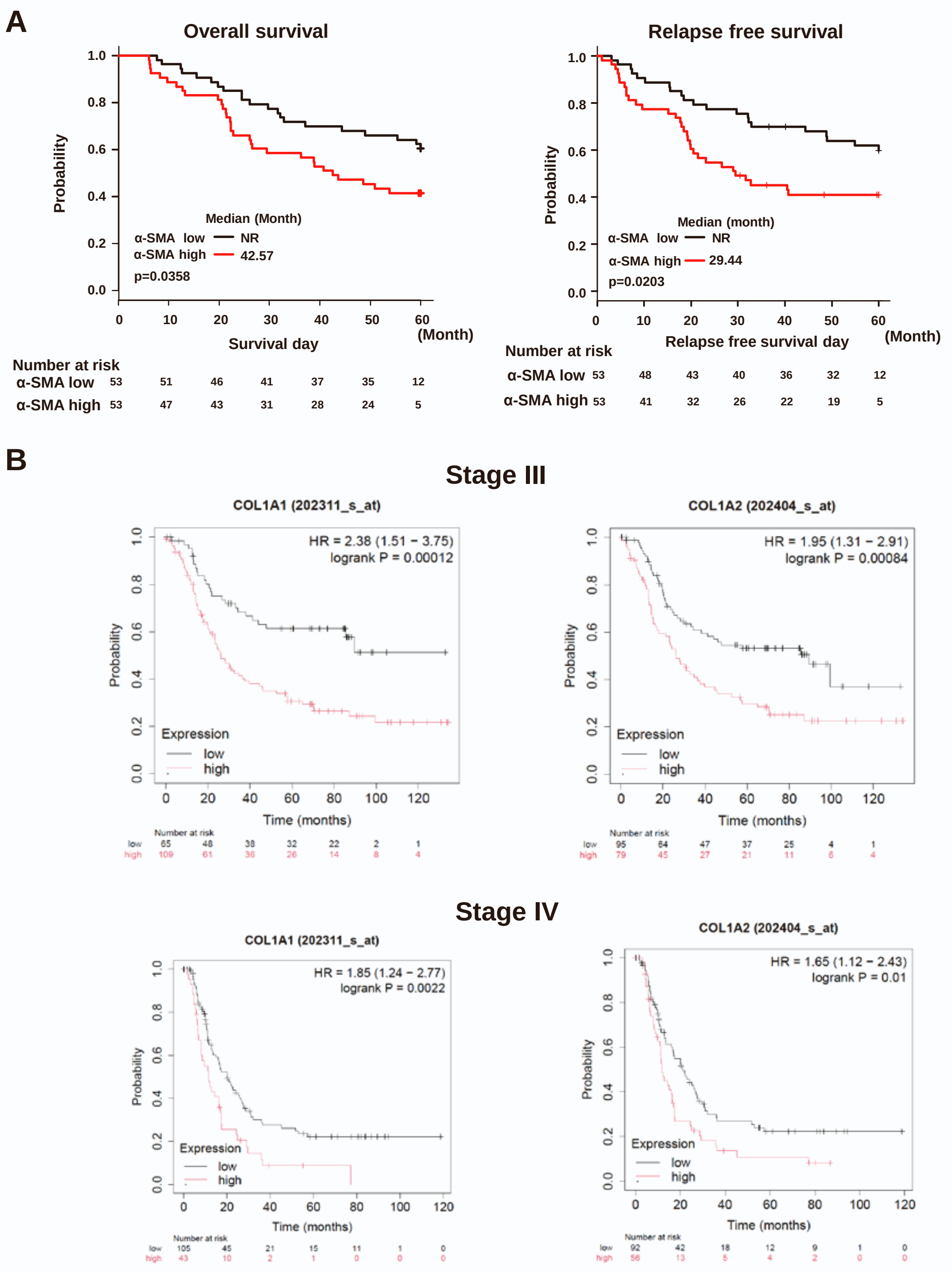

Fig. S1

A) Overall survival and relapse free survival curve according to α-SMA expression (high or low) in the primary gastric cancer. The high α-SMA expression group showed significantly worse prognosis (log rank test).

B) The relationship between the RNA expression levels of Col1A1 or Col1A2 and overall survival estimates in patients for Stage III and Stage IV gastric cancer patients was evaluated using Kaplan–Meier plotter datasets.

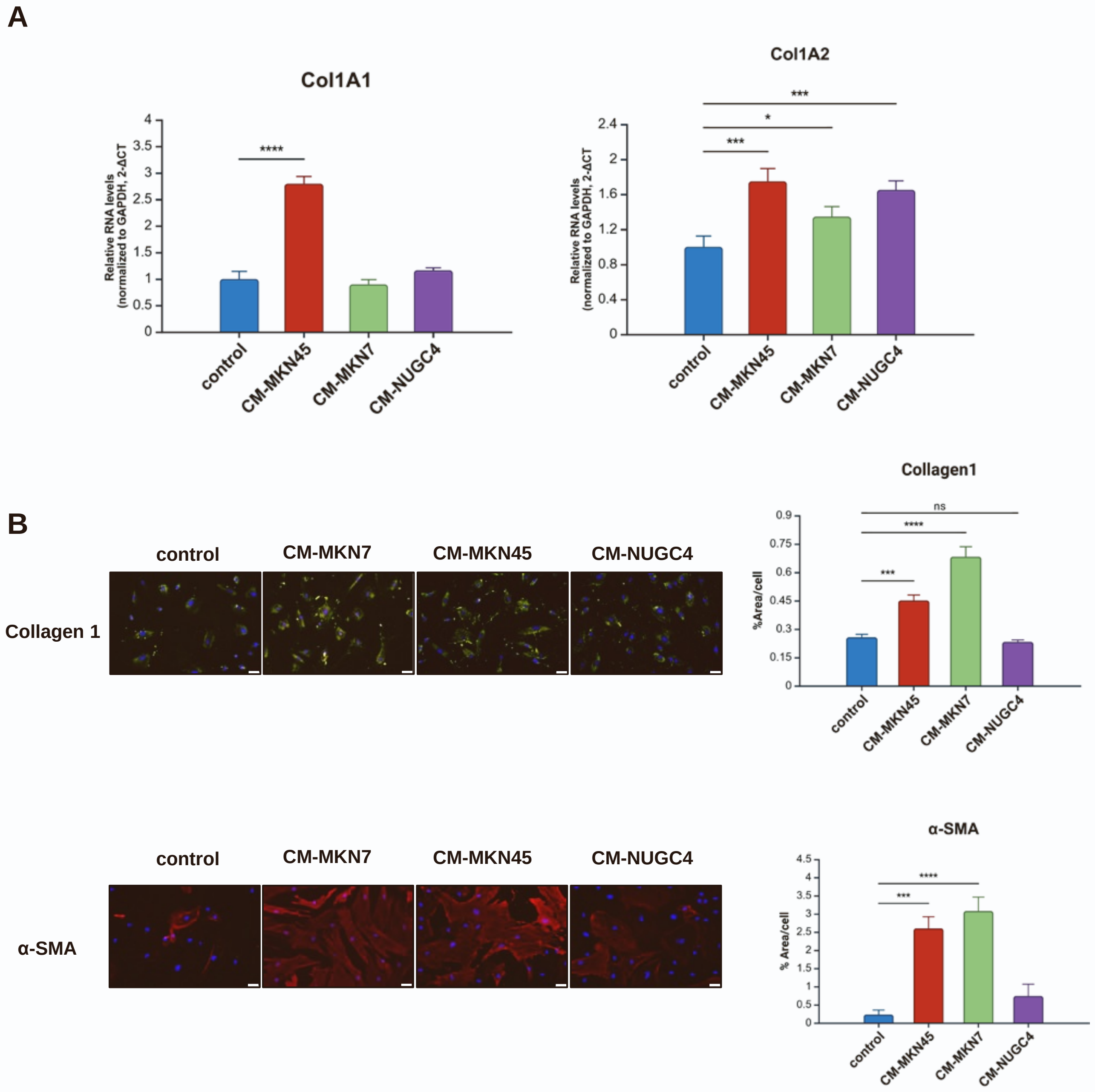

Fig. S2

The functional changes of FEF3 cells after incubation with conditioned medium of gastric cancer cells. **A)** Expression of Col1A1 and Col1A2 mRNA in FEF3 cell after incubation with condition medium (CM) of each human gastric cancer cells for 4 days. Cells were analyzed using quantitative RT-PCR analysis. Data are expressed as mean ± SD (n=3). **B)** Representative images of immunocytochemical staining of collagen1 and α-SMA in FEF3 after incubation with CM of each GC cells for 4 days. Serum-free medium (SFM) was used as a control. The area index for each staining was evaluated by Image J software. Data are expressed as mean ± SD (n=3). Scale bar, 50 μm. *$p<0.05$, ***$p<0.001$, ****$p<0.0001$.

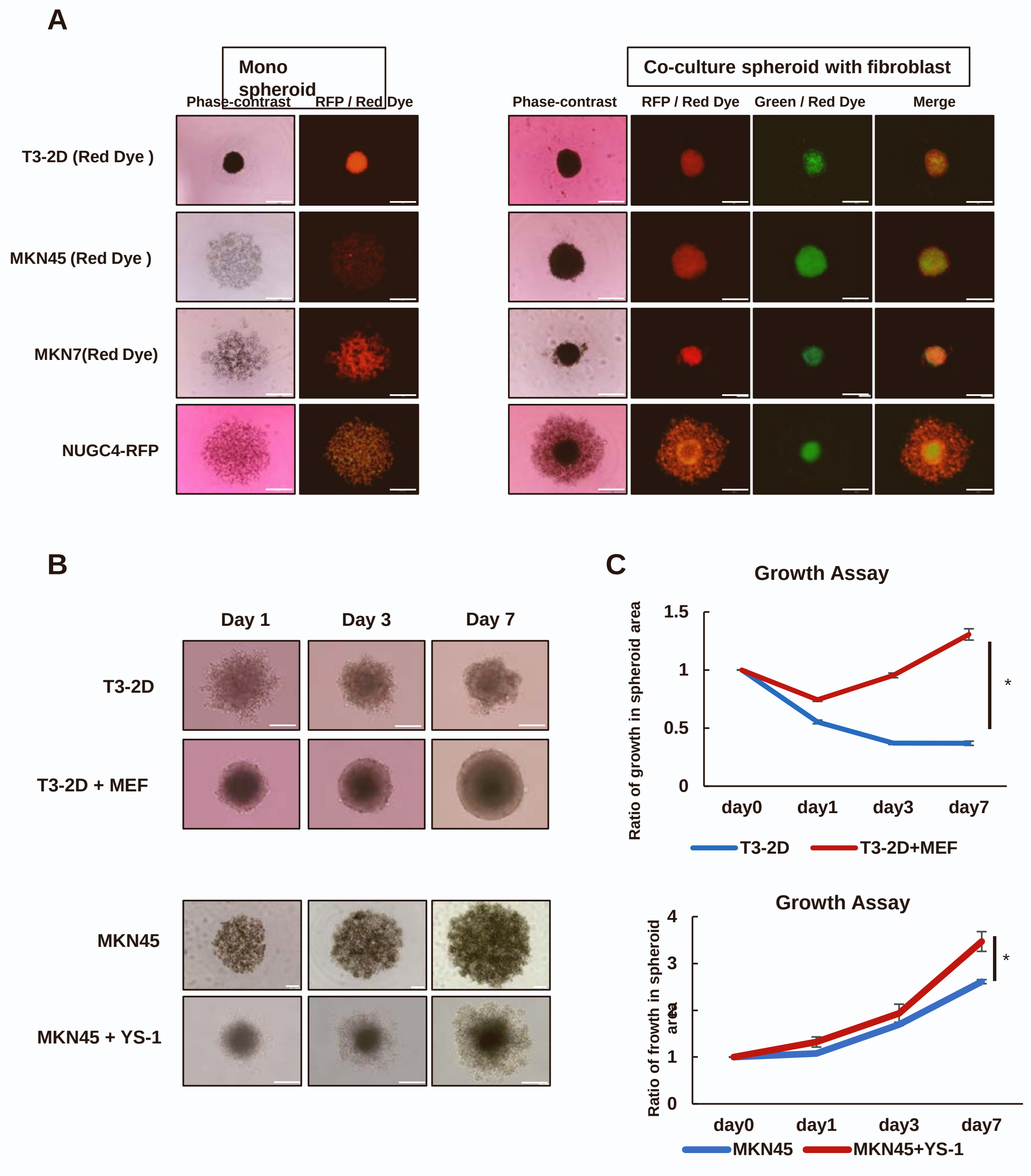

Fig. S3

Representative microscopic images of spheroids of gastric cancer cells and co-cultured with fibroblasts. **A)** Mono spheroids were cultured with 5000 gastric cancer (GC) cells and co-cultured spheroids were cultured with 5,000 GC cells and 10,000 fibroblasts for 48 hours. GC cells were stained with 10 mM CellTrackerTM Red CMTPX Dye and fibroblasts were stained with 10 mM CellTrackerTM Green CMFDA Dye. Scale bar, 200 µm. **B)** Representative microscopic images of growth of mono-spheroids or co-cultured spheroids over time for 7 days. Scale bar, 100 µm. **C)** Data are expressed as ratio of change in spheroid circumference ± SD (n = 5). Statistical significance was determined using Liner Regression. *, $P < 0.05$.

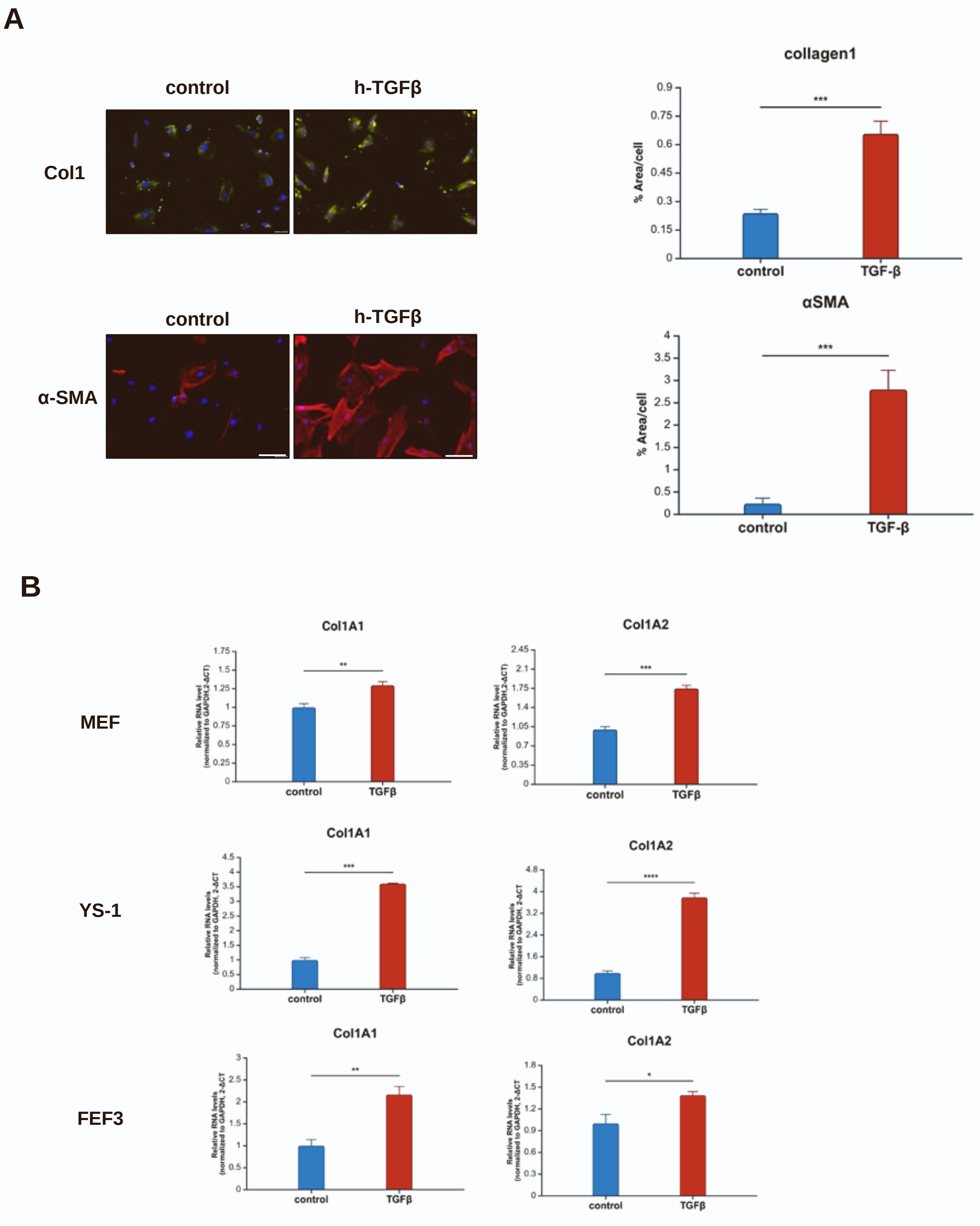

Fig. S4

The functional changes of fibroblasts after incubation with TGF-β. **A)** Representative images of immunocytochemical staining of collagen1 and α-SMA in FEF3 cell after incubation with recombinant TGF-β (10 ng/ml) for 4 days. SFM was used as a control. The area index for each staining was evaluated by Image J software. Data are expressed as mean ± SD (n=3). Scale bar, 100 μm. **B)** Expression of Col1A1 and Col1A2 mRNA in MEF, YS-1 and FEF3 cells after incubation with recombinant TGF-β (10 ng/ml or 100 ng/ml) for 4 days. Cells were analyzed using quantitative RT-PCR analysis. Data are expressed as mean ± SD (n=3). *p<0.05, **p<0.01, ***p<0.001, ****p<0.0001.

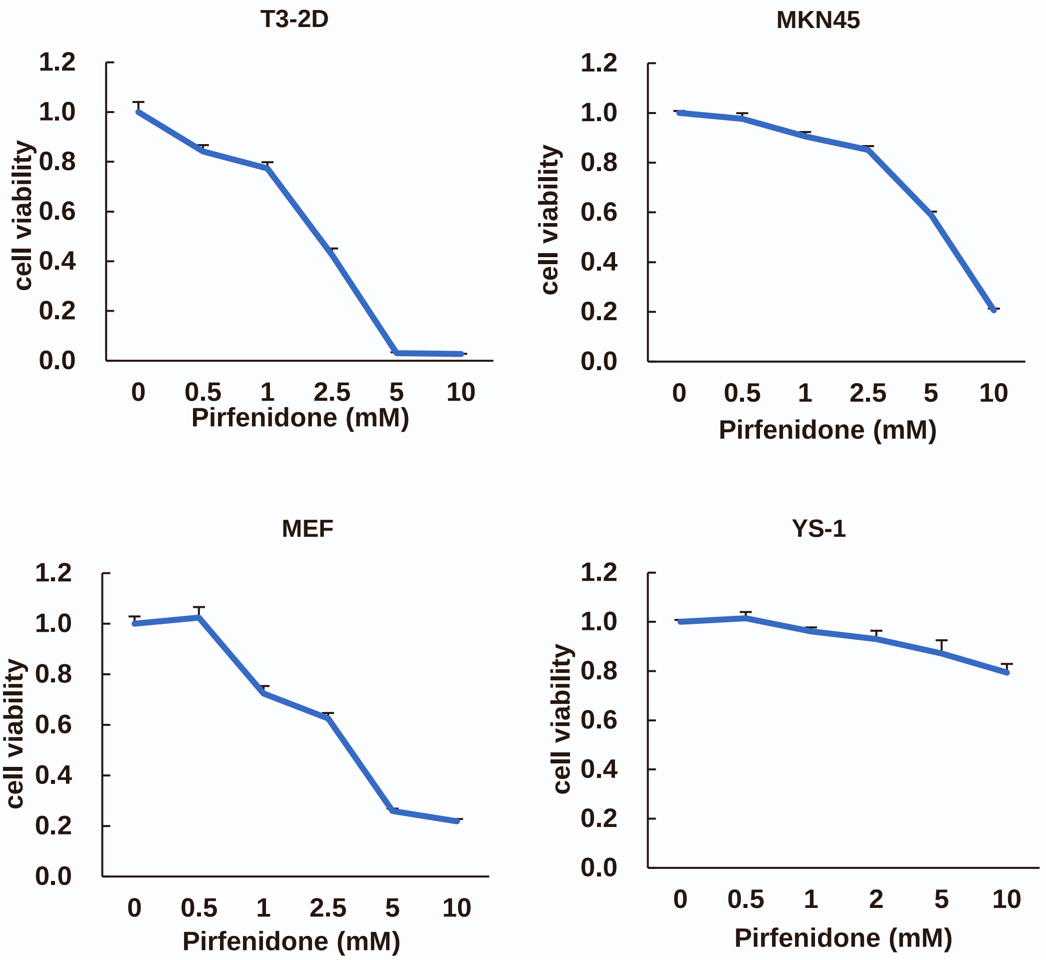

Fig. S5

Cell viability assay of gastric cancer cells and fibroblasts after treated with Pirfenidone. T3-2D, MKN45, MEF and YS-1 cells were treated with Pirfenidone at the concentration of 0, 0.5, 1, 2.5, 5 or 10 mM for 72 hours. Cell viability assessed using the XTT assays. Cell viability was calculated relative to that of the mock-treated cells, which were set as 1.0. Data are expressed as mean ± SD (n = 5).

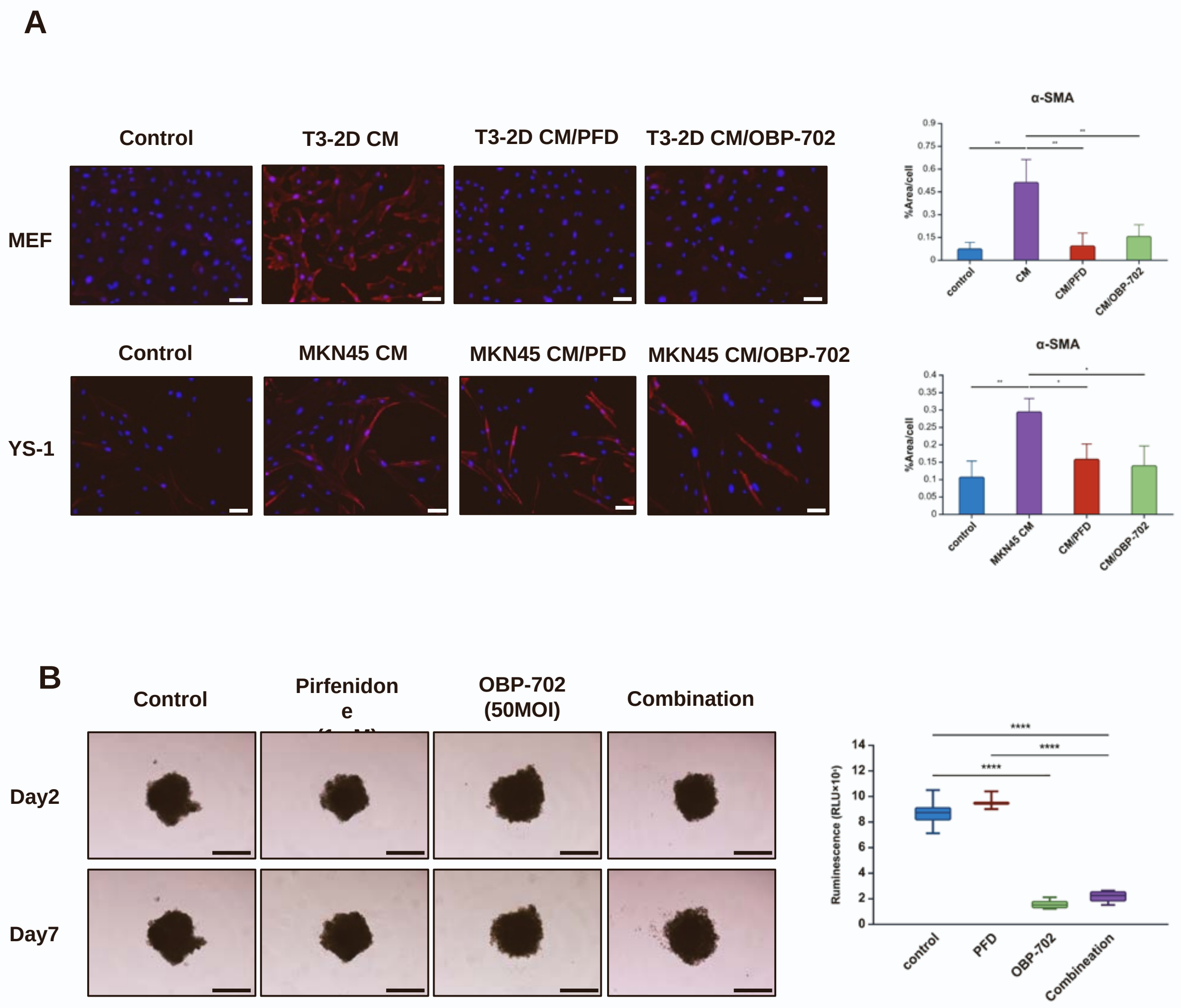

Fig. S6

The efficacy of Pirfenidone and OBP-702 on CAFs and spheroids od cancer cells and fibroblasts. **A)** Representative images of immunocytochemical staining of α-SMA in MEF and YS-1 cells after incubation with CM of T3-2D or MKN45 cells and treated with Pirfenidone (PFD) (1mM) or OBP-702 (20MOI or 50MOI) for 4 days. SFM was used as a control. The area index for each staining was evaluated by Image J software. Data are expressed as mean ± SD (n=3). Scale bar, 100 μm. **B)** Representative images of MKN45 co-cultured with YS-1 cells spheroids after treated with PFD (1mM), OBP-702 (50MOI) or the combination for 7 days. ATP cell viability assay of co-culture spheroid after treated with PFD (1mM), OBP-702 (50MOI) or the combination at 7 days after treatment. Data are expressed as mean ± SD (n = 5). *p<0.05, **p<0.01, ****p<0.0001.

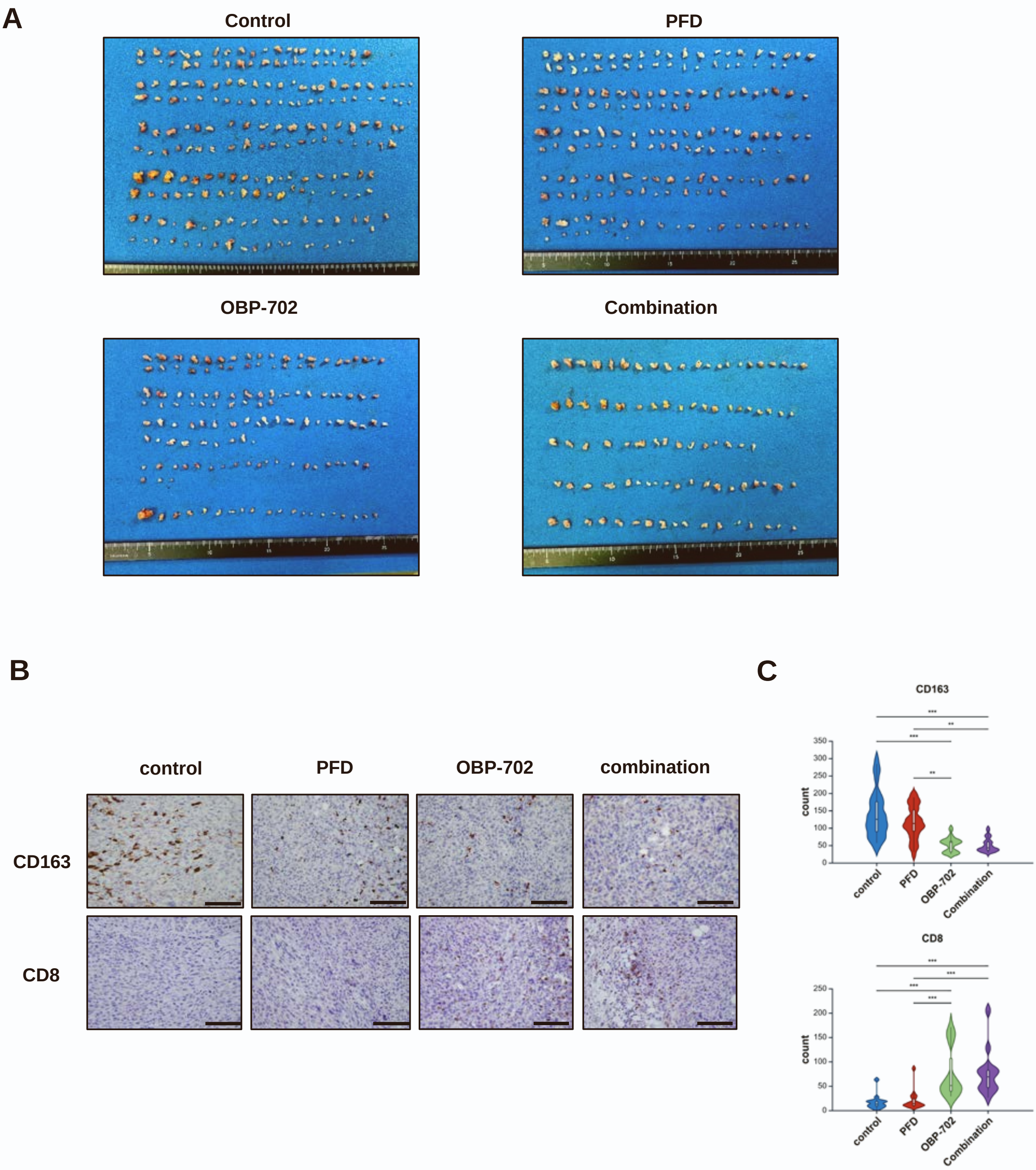

Fig. S7

Anti-tumor effects of Pirfenidone and OBP-702 on orthotopic mouse peritoneal metastasis model of T3-2D and co-injected with MEF. **A)** Macroscopic images of peritoneal nodules of in each group. **B)** Representative microscopic images of CD8 and CD163 staining of peritoneal nodules in each group. Scale bar, 100 μm. **C)** The mean cell numbers of CD8 and CD163 expression in peritoneal tumors treated with each treatment. The mean cell numbers were calculated from three selected fields in each mouse, from a total of five mice per group. Data are expressed as the mean ± SD (n = 5). *p<0.05, **p<0.01, ***p<0.001, ****p<0.0001.

Table. S1

Patients' demographics and pathological characteristics of 106 advanced gastric cancer patients.

		Collagen high (n=53)	Collagen low (n=53)	p-value
**Background**	Age (median, years)	65	74	<0.001
	Sex; M: F	40: 13	37: 16	n.s
	BMI (median)	22.3 (20.7-24.0)	22.3 (19.5-25.6)	n.s
**Pathological diagnoses**	SS: SE	20: 33	25: 28	n.s
	pStage[a]; II: III: IV	20: 24: 9	28: 18: 7	n.s
	Histological type; Intestinal: diffuse	14: 39	29: 24	0.0053
	ly; 0 or 1: 2 or 3	6: 47	17: 36	0.017
	v;0 or 1: 2 or 3	17: 36	19: 34	n.s
	Lymph node metastasis	45	35	0.041
	αSMA (median)	2.601 (1.93-3.65)	2.09 (1.54-2.61)	0.0026
**Recurrence**	All	32	15	0.0016
	Peritoneal metastasis	23	8	0.0025
	Liver metastasis	5	7	n.s

[a]Tumor stage is classified by Japanese Classification of Gastric Carcinoma, 3rd English edition

Table. S2

Primer sequences for qRT-PCR.

**Primers for qRT-PCR**	
**Gene**	**Primer sequence**
GAPDH (human)	Forward: CTGCACCACCAACTGCTTAG<br>Reverse: GTCTTCTGGGTGGCAATGAT
Col1A1 (human)	Forward: GATTCCCTGGACCTAAAGGTGC<br>Reverse: AGCCTCTCCATCTTTGCCAGCA
Col1A2 (human)	Forward: CCTGGTGCTAAAGGAGAAAGAGG<br>Reverse: ATCACCACGACTTCCAGCAGGA
ACTA2 (human)	Forward: CTATGCCTCTGGACGCACAACT<br>Reverse: CAGATCCAGACGCATGATGGCA
